# Early‑life head circumference is causally associated with adult executive function through cortical surface area expansion

**DOI:** 10.1016/j.dcn.2026.101805

**Published:** 2026-08-29

**Authors:** Junjiao Feng, Qingjing Jia, Dekang Wang, Feng Liu

**Affiliations:** a Faculty of Psychology, Tianjin Normal University, Tianjin 300387, China; b Department of Radiology and Tianjin Key Laboratory of Functional Imaging & Tianjin Institute of Radiology, Tianjin Medical University General Hospital, Tianjin 300052, China

**Keywords:** Early-life head circumference, Cortical morphology, Executive functions, Genetic correlations, Mendelian randomization

## Abstract

Executive functions (EFs) are heritable higher‑order cognitive abilities, with a common factor (cEF) capturing shared variance across tasks. Whether early brain growth contributes to adult EF (and through which pathways) remains unclear. Using GWAS summary statistics, we applied linkage disequilibrium score regression, bidirectional two-sample Mendelian randomization (MR), and multivariable MR to investigate the genetically predicted influence of infant head circumference (HC) on adult cEF and the mediating role of cortical morphology. Infant HC, but not birth HC, showed a significant positive genetic correlation and a unidirectional genetically predicted effect on adult cEF. Total and regional cortical surface area (SA) in frontal, cingulate, and temporal regions exhibited significant genetic correlations and genetically predicted effects on adult cEF, with MVMR confirming that these regional effects remained significant after mutual adjustment. Infant HC showed strong genetic correlations and directional associations with the SA of these cEF‑related regions. Two‑step MR mediation analysis indicated that total and regional SA (anterior cingulate, superior frontal, superior temporal) are consistent with a partial mediating role in the genetically informed pathway from infant HC to adult cEF. Reverse mediation analyses further suggested a similar mediating role for infant HC in the pathway from cortical SA to adult cEF. Together, these results point to bidirectional mediation between infant HC and cortical SA, supporting a genetic‑developmental pathway in which early-life HC and cortical structure interact to shape lifelong executive capacity.

## 1 Introduction

Executive functions (EFs) comprise a set of higher-order cognitive processes, including working memory, inhibitory control, and cognitive flexibility, that support goal-directed behavior across the lifespan (Diamond, 2013, Karr et al., 2022, Miyake et al., 2000, Naveh-Benjamin and Cowan, 2023, Toba et al., 2024). Individual differences in EFs are strongly associated with educational attainment (Diamond, 2013, Folker et al., 2025, Turnbull et al., 2024), occupational success (Diamond, 2013, Folker et al., 2025, Zelazo et al., 2024), mental health (Burghart et al., 2024, Diamond, 2013, Folker et al., 2025, Fontaine et al., 2026, Stucke and Doebel, 2024, Yang et al., 2022), and physical well-being (Diamond, 2013, Wheeler et al., 2020). Moreover, twin and molecular genetic studies indicate that EFs are moderately to highly heritable (Freis et al., 2022, Friedman et al., 2008, Miyake and Friedman, 2012, Polderman et al., 2015).

Latent variable studies have shown that EF exhibits both unity and diversity, with a common executive function (cEF) factor capturing shared variance across EF tasks (Feng et al., 2022, Friedman and Miyake, 2017, Miyake and Friedman, 2012, Miyake et al., 2000). This cEF factor reflects the ability to maintain and use goal-relevant information to bias ongoing processing, which is a core capacity required across diverse EF tasks (Botvinick et al., 2001, Friedman and Miyake, 2017, Miller and Cohen, 2001, Reineberg et al., 2022). Notably, cEF is the primary source of the predictive power of EF for clinically and socially relevant outcomes (Hatoum et al., 2023, Herd et al., 2014, Miyake and Friedman, 2012, Romer and Pizzagalli, 2021, Snyder et al., 2015, Tubbs et al., 2025, Yan et al., 2026). Therefore, understanding the developmental origins of adult EF, particularly the cEF factor, remains a key challenge. While the heritability of EF is well established, it remains unclear whether the link between early neurodevelopment and adult cognitive capacity reflects causal developmental pathways that actively influence adult executive function, shared genetic influences that persist across the lifespan, or residual confounding from socioeconomic and environmental factors. Distinguishing among these possibilities is essential for identifying early developmental windows for intervention and for understanding the neurobiological foundations of executive control.

Brain structure represents a critical intermediate phenotype linking genes to cognition. Large-scale neuroimaging consortia have demonstrated that cortical surface area (SA) and thickness (TH) are themselves highly heritable traits, governed by partly distinct genetic architectures (Grasby et al., 2020, Hofer et al., 2020, Panizzon et al., 2009, Strike et al., 2018, van der Meer et al., 2020, Warrier et al., 2023). Importantly, association cortices within frontal and temporal regions, which are key substrates of EFs, undergo prolonged maturation and exhibit substantial interindividual variability (Dong et al., 2024, Gogtay et al., 2004, Keller et al., 2023, Mills et al., 2021, Sydnor et al., 2023). Although prior studies have identified phenotypic associations between brain morphology and cognitive ability (Chou et al., 2025, Cox et al., 2019, Deary et al., 2010, Li et al., 2025, Miranda et al., 2025, Moodie et al., 2025), it remains unclear whether these relationships reflect shared genetic influences (Chen et al., 2025, Lett et al., 2020), causal developmental pathways (Korologou-Linden et al., 2023), or residual confounding (Thomas and Coecke, 2023, van Drunen et al., 2024).

To move beyond these adult-centric structural associations, and to test whether the link between brain structure and EF has its origins early in life, we turn to an earlier developmental stage. A developmental account of the brain-cognition association predicts that early-life measures of brain size should be linked to adult cortical structure and, through it, to adult executive function. This is because the cerebral cortex undergoes its most rapid growth during the first two years of life (Gilmore et al., 2018, Gogtay et al., 2004, Huang et al., 2022), and individual differences in early growth trajectories may persist into adulthood (Fjell et al., 2015, Walhovd et al., 2016). Early-life head circumference (HC) is widely used as a proxy for early brain size and growth trajectory (Haworth et al., 2019, Taal et al., 2012, the Early Growth Genetics, C, 2022). Longitudinal studies suggest that early postnatal head growth is associated with later cognitive outcomes (Fu et al., 2023, Hagenaars et al., 2016, Horner et al., 2025, Lee et al., 2019, Selvanathan et al., 2022, Zhu et al., 2022), and genome-wide association studies (GWAS) have identified genetic variants influencing HC at birth and in infancy (Taal et al., 2012, the Early Growth Genetics, C, 2022). This leads to the specific prediction that genetic factors influencing early-life HC contribute to adult cEF through their effect on cortical structure. However, this prediction has remained untested, in part because observational studies cannot easily distinguish between genetic confounding and genuine developmental influences, and because the neuroanatomical pathways that might mediate such a relationship have not been systematically examined. Addressing this gap is important for moving beyond purely correlational evidence toward a better understanding of whether and how early brain development may shape adult cognitive capacity.

Recent methodological advances enable more rigorous investigation of these questions. Linkage disequilibrium score regression (LDSC) allows estimation of genome-wide genetic correlations using summary statistics (B. Bulik-Sullivan et al., 2015; B. K. Bulik-Sullivan et al., 2015; Finucane et al., 2015; Zhao et al., 2025), while Mendelian randomization (MR) leverages genetic variants as instrumental variables (IVs) to infer causal effects between traits (Bowden et al., 2015, Chen et al., 2024, Davey Smith and Hemani, 2014, Lee and Lee, 2025, Sanderson et al., 2022, Sanderson et al., 2021, Zhao et al., 2026). Large-scale GWAS datasets for early-life HC (Vogelezang et al., 2022), cortical morphology (Grasby et al., 2020), and cEF (Hatoum et al., 2023) now provide unprecedented statistical power to examine developmental pathways spanning infancy to adulthood. However, an integrated genetic framework linking early-life HC, cortical structural organization, and cEF has yet to be systematically tested.

In the present study, we address this gap by combining LDSC, bidirectional two-sample MR, multivariable MR (MVMR), and two-step MR mediation analyses to directly test this developmental hypothesis. Specifically, we ask whether genetic factors influencing early-life HC are genetically correlated with and directionally associated with adult cEF, and whether cortical structure is consistent with a mediating role in this relationship. We also examine whether this mediation is bidirectional, given the possible bidirectional associations between infant HC and cortical SA. If such a pathway exists, it has potential clinical and public health implications. HC is a simple, non-invasive, and routinely collected pediatric measure that could serve as an early indicator of risk for cognitive difficulties, enabling earlier identification of children who may benefit from cognitive support. Moreover, understanding the mediating cortical mechanisms could inform biologically targeted interventions during critical windows of infancy and early childhood

## 2 Methods

### 2.1 Study design

**Fig. 1:**
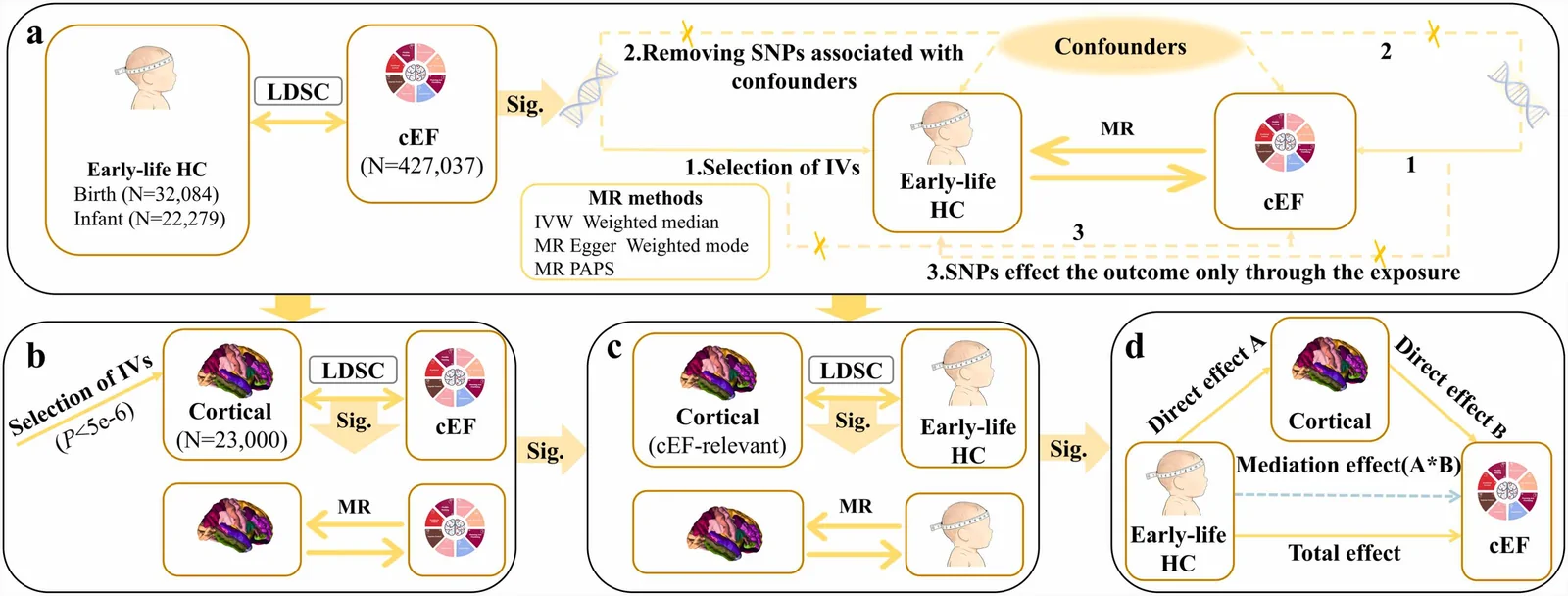
**Study design flowchart.** All analyses are based on publicly available GWAS summary statistics from previous studies (see Methods). The study was conducted in four sequential stages integrating LDSC, bidirectional two‑sample MR, MVMR and two-step MR mediation. **(a)** LDSC estimated genetic correlations between early-life HC and adult cEF, followed by bidirectional MR to determine directionality. **(b)** LDSC assessed genetic correlations between adult cEF and 70 cortical structural phenotypes (SA and TH); bidirectional MR was subsequently performed only for those with significant genetic correlations. **(c)** LDSC evaluated genetic correlations between infant HC and the cortical structural phenotypes that showed genetically predicted effects on adult cEF in stage (b); bidirectional MR further examined directional relationships between infant HC and these phenotypes. **(d)** Two‑step MR mediation analyses tested whether these cortical phenotypes are consistent with a mediating role in the pathway from early-life HC to adult cEF. **Abbreviations:** LDSC, linkage disequilibrium score regression; MR, Mendelian randomization; HC, head circumference; SA, surface area; TH, thickness; cEF, common executive function; IVW, inverse‑variance weighted; MR-PRESSO, Mendelian randomization Pleiotropy Residual Sum and Outlier.

We employed a combination of LDSC and two-sample MR to systematically investigate the genetically predicted effect of early-life HC (both at birth and during infancy), on individual differences in adult cEF, and to explore potential cortical morphology mediators of this relationship. First, LDSC estimated the genetic correlation between HC at birth (birth HC), HC during infancy (infant HC), and adult cEF. Bidirectional two-sample MR was then conducted to assess the directional nature of these associations. To identify potential neuroanatomical substrates underlying this genetically informed pathway, we performed LDSC to systematically evaluate genetic correlations between adult cEF and 70 cortical structural phenotypes, including total cortical SA, mean cortical TH, and bilateral regional measures from 34 cortical regions defined according to the Desikan–Killiany atlas. Cortical structural phenotypes showing significant genetic correlations with adult cEF were subsequently carried forward to bidirectional MR analyses. Next, we examined whether these cortical structural phenotypes (SA and TH) that showed genetically predicted associations with cEF were themselves genetically correlated with early-life HC. Where significant genetic correlations were observed, bidirectional causal relationships were tested using two‑sample MR. Finally, for significant causal associations, two‑step MR mediation analysis was performed to formally evaluate whether cortical structural phenotypes (SA and TH) are consistent with a mediating role in the pathway from early HC to adult cEF. The study flowchart is presented in Fig. 1.

### 2.2 Data sources

#### 2.2.1 GWAS summary data for early-life HC

Summary statistics for early-life HC were obtained from the Early Growth Genetics (EGG) Consortium (https://egg-consortium.org/). Data were derived from two developmental time points at birth and during infancy (defined as the measurement closest to 18 months within a 6- to 30-month window). The analysis of birth HC included up to 32,084 children from 22 studies, whereas the infant HC analysis included up to 22,279 children from 21 studies. All summary statistics were derived from participants of European ancestry (Vogelezang et al., 2022). Detailed cohort characteristics are provided in Supplementary Table S1.

#### 2.2.2 GWAS summary data for cortical structural phenotypes

Summary statistics for cortical SA and TH were obtained from a cortical morphometric meta‑analysis by the ENIGMA Consortium (Grasby et al., 2020). The dataset comprised structural MRI scans from multiple cohorts worldwide. Using the Desikan–Killiany atlas, 34 regions per hemisphere were defined, from which total SA, mean TH, and bilateral regional values were extracted, yielding 70 cortical structural phenotypes. For the present analysis, we restricted the sample to European ancestry and excluded UK Biobank participants (to avoid sample overlap with the cEF GWAS, see below), resulting in a final analytical sample of approximately 23,000 participants. Full cohort descriptions are provided in Supplementary Table S2. The original GWAS estimates were provided as unstandardized betas and standard errors. We standardized these estimates by dividing each unstandardized beta and its standard error by the standard deviation of the corresponding phenotype in the original GWAS sample.

#### 2.2.3 GWAS summary data for adult cEF

Summary statistics for the cEF factor were obtained from a study by Hatoum. et al. (2023). In that study, a phenotypic cEF factor score was derived from five cognitive tasks in the UK Biobank (Trail Making Test, Symbol Digit Substitution, Backward Digit Span, Prospective Memory, and Pairs Matching), with prior validation supporting their shared executive components. Confirmatory factor analysis generated individual cEF factor scores, which were used in a genome-wide association analysis of 427,037 individuals of European ancestry with complete genetic and phenotypic data.

#### 2.2.4 Genetic correlation analyses

We estimated genetic correlations between early-life HC, cortical structural phenotypes (SA and TH), and adult cEF using LDSC (B. Bulik-Sullivan et al., 2015) based on publicly available GWAS summary statistics. We applied precomputed linkage disequilibrium (LD) scores from the 1000 Genomes Project European reference panel and restricted all analyses to HapMap3 single‑nucleotide polymorphisms (SNPs). Pairwise genetic correlations were considered significant after Bonferroni correction (Curtin and Schulz, 1998) (*P* < 0.05), and only significant phenotype pairs were retained for subsequent MR analyses.

### 2.3 MR analyses

#### 2.3.1 Selection of genetic instruments

We conducted two‑sample MR using summary‑level data, with SNPs serving as IVs for each exposure. Valid MR relies on three core assumptions (Emdin et al., 2017). First, the genetic variants must be strongly associated with the exposure (relevance). Second, they must not be associated with any known confounders (independence). Third, they must influence the outcome solely through the exposure, with no direct or alternative pathways (exclusion restriction).

To satisfy the relevance assumption, we selected SNPs based on their strength of association with the exposure. A genome‑wide significance threshold of *P* < 5 × 10^−8^ was applied when the exposure was adult cEF. For early-life HC or cortical structural phenotypes (SA and TH), the threshold was relaxed to *P* < 5 × 10^−6^ because an insufficient number of SNPs met the stricter cutoff. We calculated the F‑statistic for each SNP and retained only those with F > 10 to minimize weak‑instrument bias (Bowden et al., 2019, Burgess et al., 2011). Independent SNPs were identified through LD clumping (r² < 0.001, distance > 10 Mb) using the 1000 Genomes European data as the reference panel.

To address the independence assumption, we queried the PhenoScanner database (Kamat et al., 2019, Staley et al., 2016) and the NHGRI‑EBI GWAS catalog (https://www.ebi.ac.uk/gwas/docs/file-downloads/) for previously reported associations (*P* < 5 ×10^−8^) of each instrument with potential confounders, including educational attainment, psychiatric disorders, intelligence, smoking, alcohol use, and sleep traits. SNPs showing such associations were removed.

For the exclusion‑restriction assumption, we excluded SNPs that were strongly associated with the outcome (*P* < 5 ×10^−6^) in the respective outcome GWAS. Palindromic SNPs were discarded during harmonization of exposure and outcome datasets. Finally, we applied the MR Pleiotropy RESidual Sum and outliers (MR-PRESSO) test (Verbanck et al., 2018) to detect and remove outlier SNPs that might indicate horizontal pleiotropy. The remaining set of SNPs was used for all primary MR analyses. Full lists of IVs for each analysis are provided in Supplementary Table S12.

#### 2.3.2 Univariable MR

We estimated causal effects using the random‑effects inverse‑variance weighted (IVW) method (Slob and Burgess, 2020) as the primary analysis, with four complementary MR methods to account for potential heterogeneity and pleiotropy**,** these included the MR‑Egger (Bowden et al., 2015), the weighted median (Bowden et al., 2016), the weighted mode (Hartwig et al., 2017), and the robust adjusted profile score (MR‑RAPS) method (Zhao et al., 2020).

The random‑effects IVW approach combines the ratio estimates of SNP associations with the exposure and with the outcome in a meta-analytic framework while allowing for heterogeneity across variants. To assess the robustness of the IVW results and to accommodate potential violations of the IV assumptions, we applied four additional methods. MR‑Egger incorporates an intercept term to detect and adjust for directional horizontal pleiotropy, albeit with reduced statistical power. The weighted median method yields consistent estimates when at least 50% of the weight is derived from valid instruments. Similarly, the weighted mode method provides valid estimates when the largest cluster of instruments with similar causal effects comprises valid variants. MR‑RAPS accounts for both systematic and idiosyncratic pleiotropy and is particularly well-suited for analyses involving many weak instruments. Results from all five methods are reported. A genetically predicted effect was considered robust when the IVW estimate remained significant after Bonferroni correction and the direction of effect was consistent across all complementary MR methods.

#### 2.3.3 MVMR

To estimate the direct effects of correlated exposure traits on an outcome while accounting for their shared genetic architecture, we performed MVMR (Sanderson et al., 2021). Based on the relationships among the exposures identified in univariable MR analyses, we selected exposure traits that showed significant bidirectional associations with the outcome. Genetic instruments from the relevant GWASs were combined, and LD clumping was performed to ensure the independence of SNPs. SNP effects and corresponding standard errors were extracted from each exposure’s GWAS summary statistics and harmonized with the outcome GWAS information.

However, MVMR often faces greater challenges with weak instruments compared with univariable MR. Weak instrument bias in MVMR can produce estimates that are not necessarily biased toward the null and may inflate Type I error rates (Burgess et al., 2011, Sanderson et al., 2021). Therefore, we first assessed instrument strength using conditional F-statistics (Sanderson et al., 2021).

To address weak instrument bias in MVMR, we adopted a two‑step strategy. When conditional F-statistics exceeded 10, we used the conventional MVMR-IVW method (Grant and Burgess, 2021). When conditional F-statistics fell substantially below 10, we prioritized the debiased inverse-variance weighted (dIVW) estimator (Wu et al., 2025), which corrects for finite-sample bias in the conventional IVW estimator under weak instruments. Reliable estimation is indicated by dIVW condition statistics above 20, and a significant independent effect is indicated by a 95% CI excluding zero(Wu et al., 2025). For additional instrument strength diagnostics, we calculated Q‑statistics(Sanderson et al., 2019); values above 10 indicate adequate instrument strength after accounting for heterogeneity, and a Q‑valid *P* above 0.05 indicates no evidence of invalid instruments.

#### 2.3.4 Two-step MR mediation analysis

Two‑step MR mediation analysis was conducted to explore potential mediated pathways between infant HC, cortical SA, and adult cEF. In the first step, genetic instruments for early-life HC were used to estimate their effects on cortical SA and cortical TH. In the second step, genetic instruments for cortical SA and cortical TH were used to estimate their effects on adult cEF. To comprehensively assess directionality and to test alternative causal structures, bidirectional MR analyses were conducted between each relevant pair of variables, and reverse mediation pathways were also examined by swapping the roles of exposure and mediator where applicable. The indirect effect was quantified using the product‑of‑coefficients method (VanderWeele, 2016), with its standard error estimated via bootstrapping (10,000 iterations) (Preacher and Hayes, 2008). To account for multiple comparisons across the mediation pathways, we applied Bonferroni correction to the bootstrap *P* values.

### 2.4 Sensitivity analyses

To evaluate the robustness of the MR estimates, we performed several sensitivity analyses. Heterogeneity across IVs was assessed using Cochran’s Q test (Greco et al., 2015), and potential directional horizontal pleiotropy was examined with the MR‑Egger intercept test. We also report the MR-PRESSO global test results (after outlier removal) as an additional validation. Leave‑one‑out analysis (Burgess et al., 2017) was performed to determine whether the overall estimate was disproportionately influenced by any single SNP. Given the relatively relaxed threshold used for instrument selection (*P* < 5 ×10^−6^) and the insufficient number of SNPs meeting the stricter genome‑wide significance threshold (*P* < 5 ×10^−8^), we additionally assessed the robustness of our findings using a more stringent threshold of *P* < 1 × 10^−6^.

All MR analyses were conducted in R (version 4.4.1) using the TwoSampleMR package (version 0.6.6), the MVMR package (version 0.4.8), the MRPRESSO package (version 1.0), and the mr.divw package (version 0.1.0).

## 3 Results

### 3.1 Genetic correlations and putative associations between early-life HC and adult cEF

#### 3.1.1 Genetic correlations between early-life HC and adult cEF

**Fig. 2:**
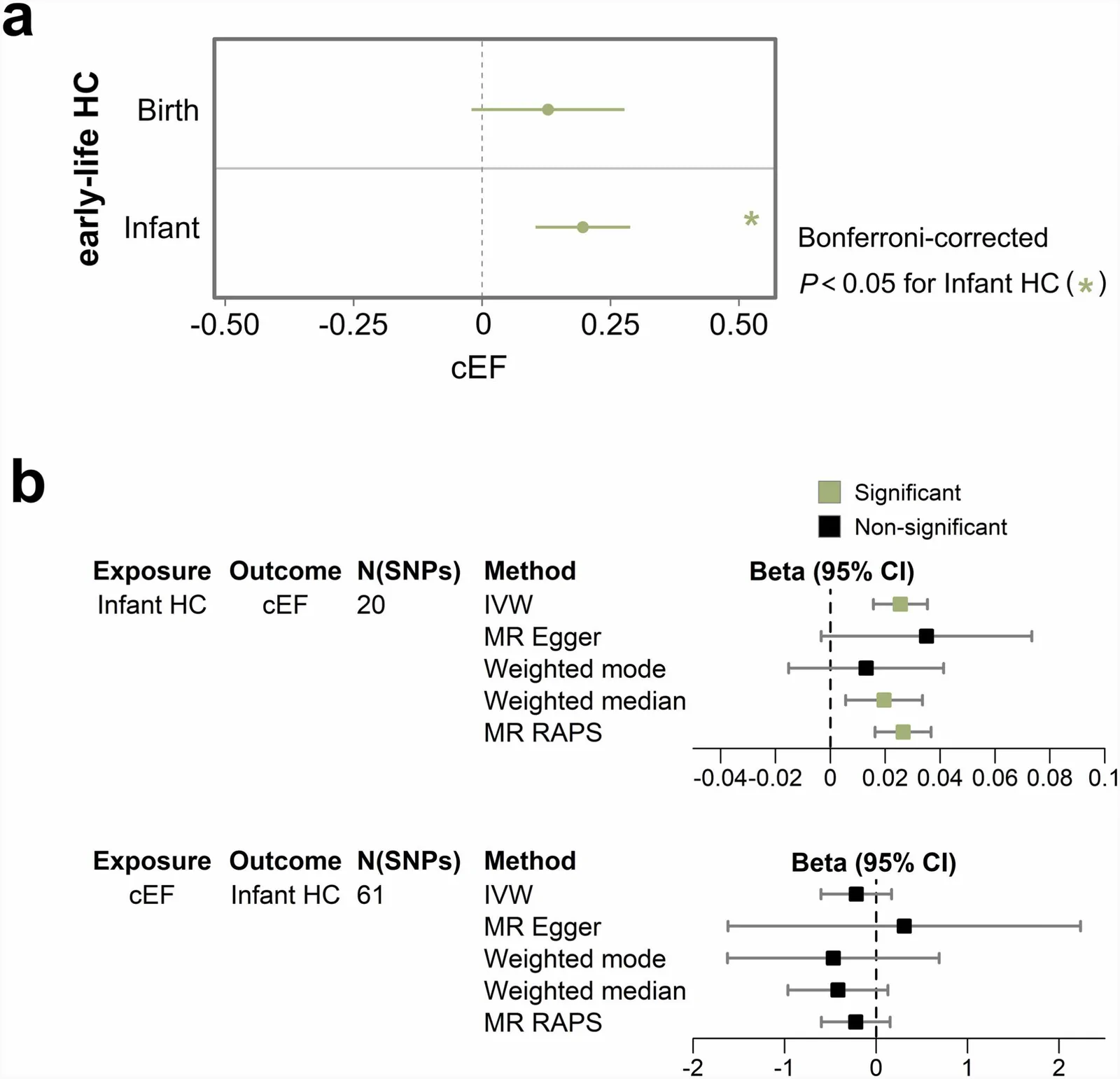
Genetic correlations and putative associations between early-life HC and adult cEF. (a) Genetic correlations between early-life HC and adult cEF. Significance was defined after Bonferroni correction (*P* < 0.025, 0.05/2). Non-significant paths are shown in gray. See Table S3 for exact results. (b) Bidirectional MR estimates between infant HC and adult cEF. Significance was defined after Bonferroni correction (*P* < 0.025, 0.05/2) and required that all of the following criteria were met: (i) IVW *P* < 0.025 with consistent effect directions across all complementary MR methods;(ii) no substantial heterogeneity after outlier removal (Cochran’s Q test *P* > 0.05); (iii) no evidence of horizontal pleiotropy (MR‑Egger intercept *P* > 0.05 and MR‑PRESSO global *P* > 0.05); and (iv) no single SNP driving the estimate (leave‑one‑out analysis). Error bars represent 95% CI. See Table S4 for exact results, and Table S13 and Figs. S1–S2 for full sensitivity analysis results. Abbreviations: See Fig. 1.

To assess the genetic overlap between early-life HC (at birth and during infancy) and adult cEF, we performed LDSC analysis. Following Bonferroni correction (*P* < 0.025, 0.05/2), we observed a significant positive genetic correlation between infant HC and adult cEF (R_g_ = 0.196, SE = 0.047, *P* = 3.01 ×10^−5^). In contrast, the genetic correlation between birth HC and adult cEF was not statistically significant (R_g_ = 0.128, SE = 0.076, *P* = 0.091). These results indicate that genetic factors influencing infant HC are shared with those underlying adult cEF, whereas no such shared genetic influence is evident at birth (Fig. 2; Table S3).

#### 3.1.2 Univariable MR analyses showed the genetically predicted effect of infant HC on adult cEF

Given the significant genetic correlation for infant HC, we next performed bidirectional two‑sample MR to examine directionality. Following Bonferroni correction (*P* < 0.025, 0.05/2), we found evidence supporting a genetically predicted effect of infant HC on adult cEF (Beta = 0.026, 95% CI: 0.016–0.035, *P* = 3.83 ×10^−7^). Conversely, the reverse MR analysis assessing the effect of adult cEF on infant HC yielded a non-significant estimate. These estimates were robustly supported by complementary MR methods, with all methods showing effect directions aligned with the IVW estimates (Fig. 2; Table S4). Together, these results suggest that the observed association is driven by a directional influence of early-life HC on subsequent EF development, rather than the reverse.

### 3.2 Genetic correlations and putative associations between cortical structural phenotypes and adult cEF

#### 3.2.1 Genetic correlations between cortical SA and adult cEF

**Fig. 3:**
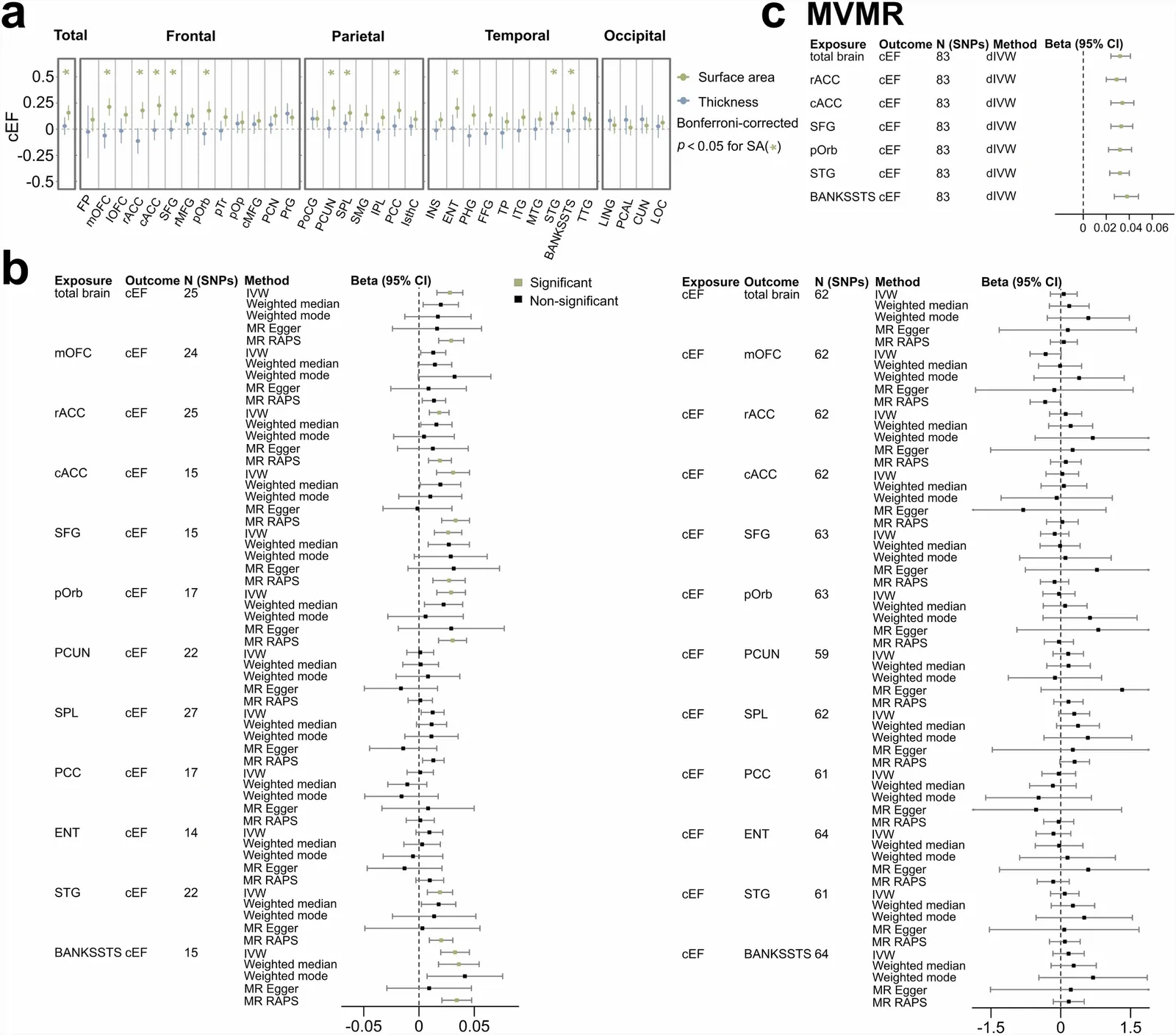
Genetic correlations and putative associations between cortical structural phenotypes and adult cEF. (a) Genetic correlations between cortical structural phenotypes and adult cEF. Significance was defined after Bonferroni correction (*P* < 7.14 ×10^−4^, 0.05/70). Error bars represent standard errors. See Table S5 for exact results. (b) Bidirectional MR estimates between cortical SA and adult cEF. Significance criteria were the same as in Fig. 2, with Bonferroni correction (*P* < 2.08 ×10^−3^, 0.05/24). Non-significant reverse estimates are shown in gray. See Table S6 for exact results, and Table S13 and Figs. S3–S10 for full sensitivity results. (c) MVMR estimates of regional SA effects on cEF. This panel shows the dIVW estimates from an MVMR model including total SA, rACC, SFG, cACC, pOrb, STG, and BANKSSTS as simultaneous exposures to estimate their independent (direct) effects on cEF. See Table S7 for exact results. Error bars represent 95% CI. Abbreviations: cEF, common executive function; SA, surface area; FP, Frontal Pole; mOFC, Medial Orbitofrontal; lOFC, Lateral Orbitofrontal; rACC, Rostral Anterior Cingulate; cACC, Caudal Anterior Cingulate; SFG, Superior Frontal; rMFG, Rostral Middle Frontal; pOrb, Pars Orbitalis; pTr, Pars Triangularis; pOp, Pars Opercularis; cMFG, Caudal Middle Frontal; PCN, Paracentral; PrG, Precentral; PoCG, Postcentral; PCUN, Precuneus; SPL, Superior Parietal; SMG, Supramarginal; IPL, Inferior Parietal; PCC, Posterior Cingulate; IsthC, Isthmus Cingulate; INS, Insula; ENT, Entorhinal; PHG, Parahippocampal; FFG, Fusiform; TP, Temporal Pole; ITG, Inferior Temporal; MTG, Middle Temporal; STG, Superior Temporal; BANKSSTS, Banks of the Superior Temporal Sulcus; TTG, Transverse Temporal; LING, Lingual; PCAL, Pericalcarine; CUN, Cuneus; LOC, Lateral Occipital.

Given the role of infant HC on adult cEF, we next investigated the neuroanatomical mediators by systematically mapping genetic overlap between cortical structure and adult cEF across 70 phenotypes (total and regional cortical SA and cortical TH for 34 parcels). Following Bonferroni correction (*P* < 7.14 ×10^−4^, 0.05/70), we identified significant positive genetic correlations between adult cEF and total cortical SA (R_g_ = 0.157, SE = 0.036, *P* = 1.55 ×10^−5^) and the SA of multiple regions within the frontal, parietal and temporal lobes (Fig. 3; Table S5). Specifically, frontal regions with significant correlations included the medial orbitofrontal cortex (mOFC, R_g_ = 0.213, SE = 0.043, *P* = 5.53 ×10^−7^), rostral anterior cingulate cortex (rACC, R_g_ = 0.179, SE = 0.041, *P* = 1.59 ×10^−5^), caudal anterior cingulate cortex (cACC, R_g_ = 0.226, SE = 0.048, *P* = 2.31 ×10^−6^), superior frontal (SFG, R_g_ = 0.141, SE = 0.040, *P* = 5.00 ×10^−4^), and pars orbitalis (pOrb, R_g_ = 0.176, SE = 0.046, *P* = 1.00 ×10^−4^). Parietal regions included the precuneus (PCUN, R_g_ = 0.200, SE = 0.041, *P* = 1.36 ×10^−6^), superior parietal lobule (SPL, R_g_ = 0.154, SE = 0.041, *P* = 2.00 ×10^−4^), and posterior cingulate cortex (PCC, R_g_ = 0.179, SE = 0.042, *P* = 1.57 ×10^−5^). Temporal regions included the entorhinal (ENT, R_g_ = 0.202, SE = 0.049, *P* = 3.94 ×10^−5^); superior temporal (STG, R_g_ = 0.150, SE = 0.036, *P* = 2.89 ×10^−5^); and the banks of the superior temporal sulcus (BANKSSTS, R_g_ = 0.153, SE = 0.044, *P* = 5.00 ×10^−4^). In contrast, genetic correlations between adult cEF and regional cortical TH measures were uniformly non-significant after multiple testing correction. Therefore, only cortical SA was carried forward to subsequent analyses.

#### 3.2.2 Univariable MR analyses showed genetically predicted effect of cortical SA on adult cEF

To determine whether the observed genetic correlations reflect potential directional influences, we performed bidirectional MR on the SA of brain regions that showed significant genetic correlations with adult cEF. Following Bonferroni correction (*P* < 2.08 ×10^−3^,0.05/24), we found evidence supporting a genetically predicted effect of the SA of several brain regions on adult cEF (Fig. 3; Table S6), including total cortical SA (Beta = 0.028, 95% CI: 0.016–0.040, *P* = 2.97 ×10^−6^), rACC (Beta = 0.018, 95% CI: 0.009–0.027, *P* = 5.90 ×10^−5^), cACC (Beta = 0.031, 95% CI: 0.016–0.046; *P* = 4.96 ×10^−5^), SFG (Beta = 0.026, 95% CI: 0.014**–**0.039, *P* = 3.30 ×10^−5^), pOrb (Beta = 0.029, 95% CI: 0.016**–**0.042, *P* = 1.09 ×10^−5^), STG (Beta = 0.019, 95% CI: 0.008**–**0.030, *P* = 1.13 ×10^−3^), and BANKSSTS (Beta = 0.033, 95% CI: 0.020**–**0.046, *P* = 8.32 ×10^−7^). These estimates were robustly supported by complementary MR methods, with all methods showing effect directions aligned with the IVW estimates. Conversely, MR analyses evaluating the effect of adult cEF on these SA measures yielded no significant estimates after correction. Thus, a unidirectional pathway consistent with a genetically predicted effect from cortical SA to adult cEF was established.

#### 3.2.3 MVMR analyses showed genetically predicted effects of cortical SA on adult cEF

Given the high correlations among regional SA measures, we next performed MVMR to determine whether these regions exert independent effects on cEF beyond the shared influence of total SA and other regional SA measures, including total SA and six regional SA measures as simultaneous exposures (Fig. 3C; Table S7). Although conditional F-statistics were below 10, the dIVW condition statistics all exceeded 20 (range: 62.42–120.97), confirming reliable estimation. Instrument validity was further supported by adequate instrument strength and no evidence of invalid instruments (Table S7). The dIVW estimates revealed significant independent effects for all exposures (all 95% CIs excluding zero). In particular, total SA (Beta = 0.032, 95% CI: 0.024**–**0.041), rACC (Beta = 0.029, 95% CI: 0.020**–**0.037), SFG (Beta = 0.033, 95% CI: 0.024**–**0.043), STG (Beta = 0.032, 95% CI: 0.023**–**0.040), cACC (Beta = 0.034, 95% CI: 0.024**–**0.044), pOrb (Beta = 0.032, 95% CI: 0.022–0.042), and BANKSSTS (Beta = 0.038, 95% CI: 0.027**–**0.048) all retained significant independent effects on cEF.

These results suggest that the observed effects of regional SA on cEF are not fully explained by shared covariance with global cortical expansion or by collinearity among regional SA measures, indicating that both global and regional surface area contribute independently to adult executive function.

### 3.3 Genetic correlations and putative associations between infant HC and cortical SA

#### 3.3.1 Genetic correlations between infant HC and cortical SA

**Fig. 4:**
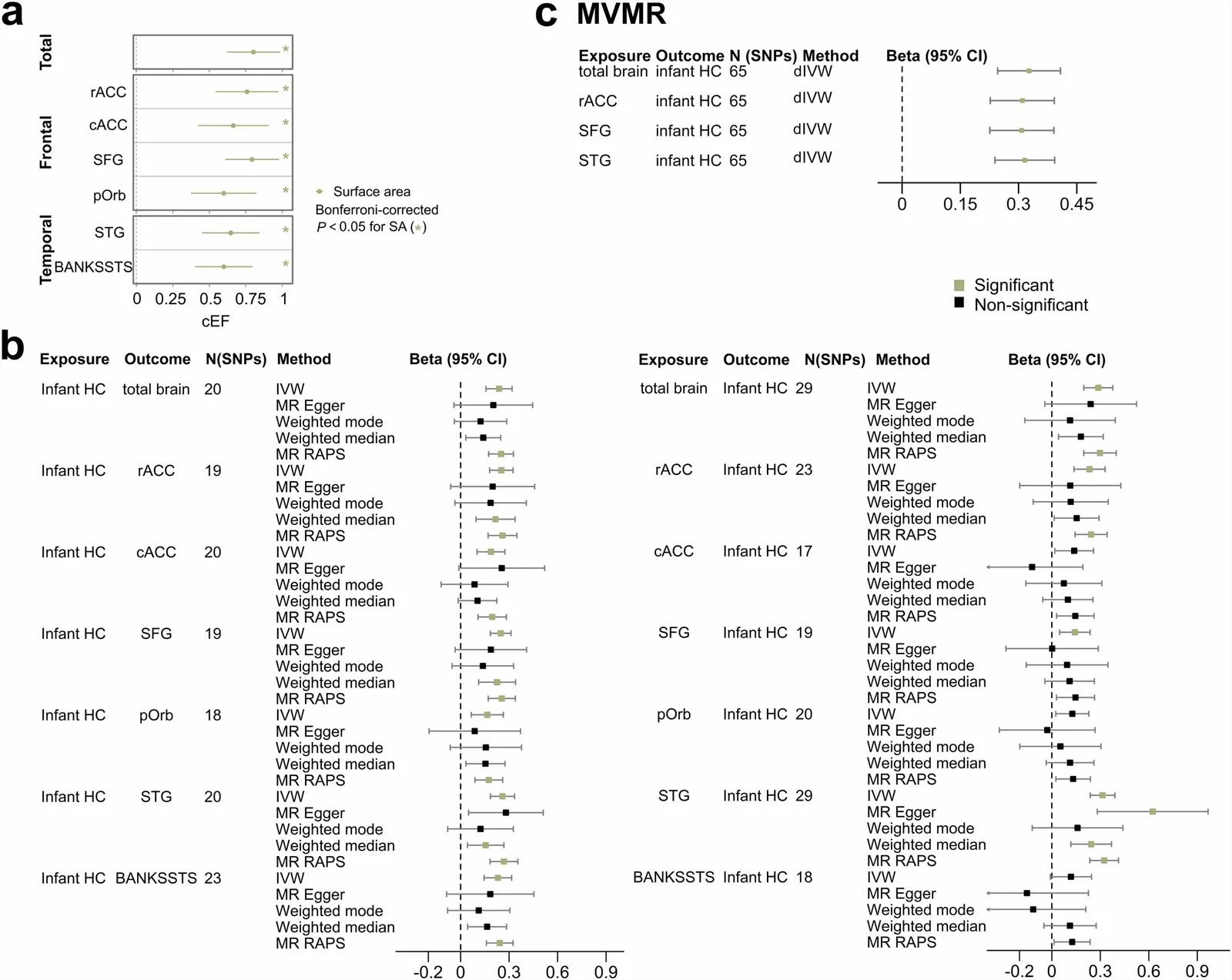
Genetic correlations and putative associations between infant HC and cortical SA. (a) Genetic correlations between infant HC and cortical SA. Significance was defined after Bonferroni correction (*P* < 7.14 ×10^⁻3^, 0.05/7)^.^ Error bars represent standard errors. see Table S8 for exact results. (b) Bidirectional MR estimates between infant HC and cEF-relevant cortical SA. Significance criteria were the same as in Fig. 2, with Bonferroni correction (*P* < 3.57 ×10^−3^, 0.05/14). Error bars represent 95% CI. See Table S9 for exact results, and Table S13 and Figs. S11–S16 for full sensitivity results. (c) MVMR estimates of regional SA effects on infant HC. This panel shows the dIVW estimates from an MVMR model including total SA, rACC, SFG, and STG as simultaneous exposures to estimate their independent effects on infant HC. See Table S10 for exact results. Error bars represent 95% CI. Abbreviations: See Fig. 3.

Having identified SA of several brain regions that show genetically predicted effects on adult cEF, we next asked whether these same regions are genetically linked to infant HC, a prerequisite for mediation. We analyzed genetic correlations between infant HC and the SA of total brain and the six regional SA that showed significant genetically predicted effects on cEF (rACC, cACC, SFG, pOrb, STG, BANKSSTS). Following Bonferroni correction (*P* < 7.14 ×10^−3^, 0.05/7), LDSC revealed robust positive genetic correlations for total cortical SA (R_g_ = 0.803, SE = 0.092, *P* = 3.21 ×10^−18^) and for each of the six regions (Fig. 4; Table S8), including rACC (R_g_ = 0.759, SE = 0.110, *P* = 5.78 ×10^−12^); cACC (R_g_ = 0.665, SE = 0.124, *P* = 7.76 ×10^−8^), SFG (R_g_ = 0.794, SE = 0.094, *P* = 4.32 ×10^−17^), pOrb (R_g_ = 0.599, SE = 0.115, *P* = 1.77 ×10^−7^), STG (R_g_ = 0.648, SE = 0.100, *P* = 1.12 ×10^−10^), and BANKSSTS (R_g_ = 0.599, SE = 0.101, *P* = 2.55 ×10^−9^). These findings indicate that genetic variants associated with infant HC are strongly linked to SA of both global and regional association cortices, suggesting a common genetic program influencing brain size from infancy.

**Fig. 5:**
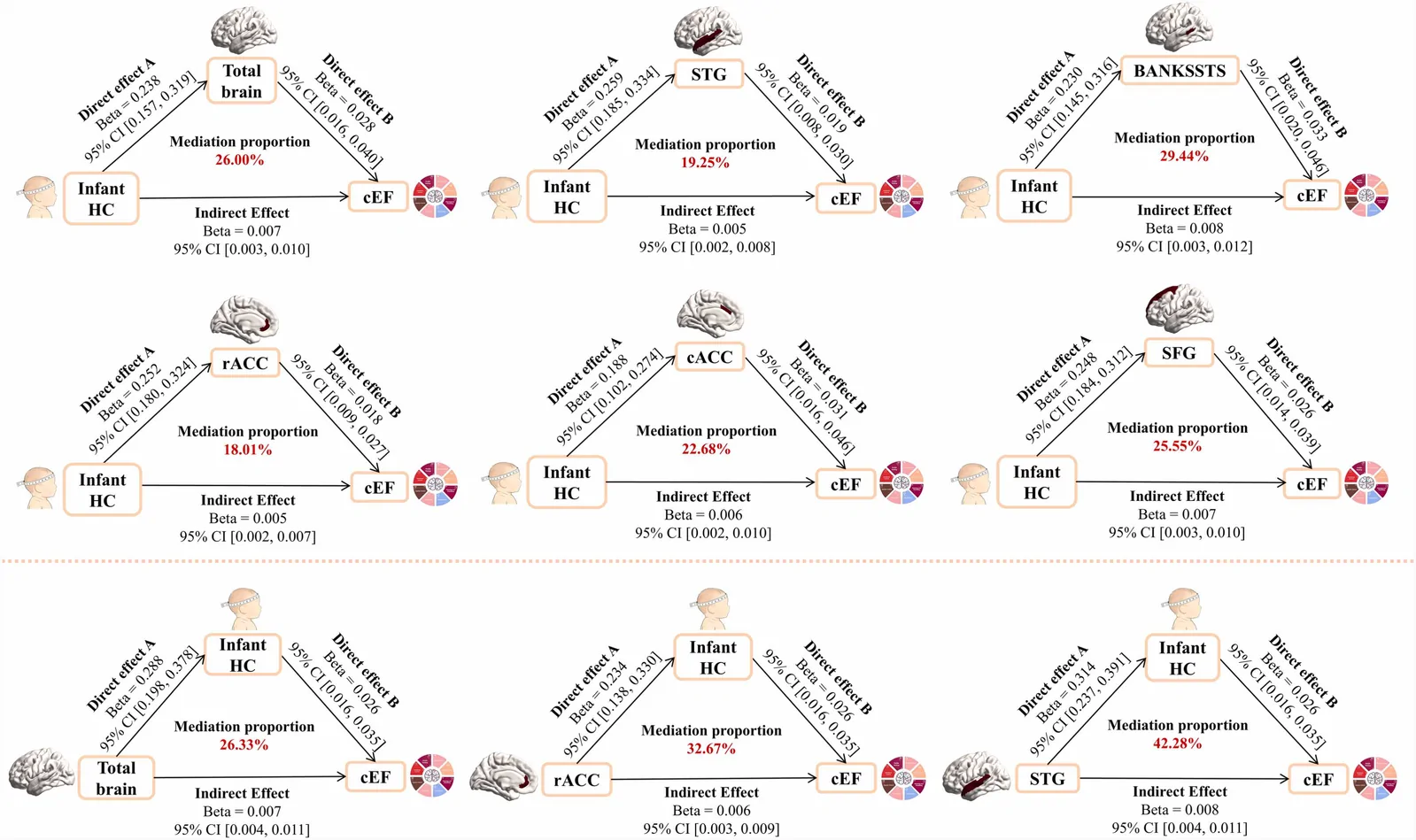
Mediation analyses among infant HC, cortical SA and adult cEF. Significance was defined after Bonferroni correction (*P* < 4.55 ×10^−3^, 0.05/11). Error bars represent standard errors. See Table S11 for exact results. Abbreviations: See Fig. 3.

#### 3.3.2 Bidirectional MR analyses of genetically predicted effects of infant HC and cortical SA

We next conducted bidirectional two‑sample MR to examine genetically inferred directional relationships between infant HC and the SA of the seven regions identified above. Following Bonferroni correction (*P* < 3.57 ×10^−3^, 0.05/14), we observed significant directional relationships (Fig. 4; Table S9). Genetic predisposition to larger infant HC increased SA of total brain (Beta = 0.238, 95% CI: 0.157**–**0.319, *P* = 8.44 ×10^−9^), rACC (Beta = 0.252, 95% CI: 0.180–0.324, *P* = 8.65 ×10^−12^), cACC (Beta = 0.188, 95% CI: 0.102**–**0.274, *P* = 1.85 ×10^−5^), SFG (Beta = 0.248, 95% CI: 0.184**–**0.312, *P* = 3.31 ×10^−14^), pOrb (Beta = 0.165, 95% CI: 0.066**–**0.265, *P* = 1.13 ×10^−3^), STG (Beta = 0.259, 95% CI: 0.185**–**0.334, *P* = 1.03 ×10^−11^), and BANKSSTS (Beta = 0.230, 95% CI: 0.145**–**0.316, *P* = 1.13 ×10^−7^). These estimates were robustly supported by complementary MR methods, with all methods showing effect directions aligned with the IVW estimates. Reciprocally, SA of total brain (Beta = 0.288, 95% CI: 0.198**–**0.378, *P* = 3.38 ×10^−10^), rACC (Beta = 0.234, 95% CI: 0.138**–**0.330, *P* = 1.86 ×10^−6^), SFG (Beta = 0.143, 95% CI: 0.048**–**0.237, *P* = 3.08 ×10^−3^), and STG (Beta = 0.314, 95% CI: 0.237**–**0.391, *P* = 9.99 ×10^−16^) also showed significant genetically predicted effects on infant HC.

#### 3.3.3 MVMR analyses showed genetically predicted effects of cortical SA on infant HC

Given the high correlations among regional SA measures and the bidirectional associations observed in univariable MR, we next performed MVMR to determine whether these regions exert independent effects on infant HC beyond the shared influence of total SA and other regional SA measures, including total SA, rACC, SFG, and STG as simultaneous exposures (Fig. 4C; Table S10). Although conditional F-statistics were below 10, the dIVW condition statistics all exceeded 20 (range: 101.86–133.20), confirming reliable estimation. Instrument validity was further supported by adequate instrument strength and no evidence of invalid instruments (Table S10). The dIVW estimates revealed significant independent effects for all exposures (all 95% CIs excluding zero). In particular, total SA (Beta = 0.327, 95% CI: 0.246–0.408), rACC (Beta = 0.310, 95% CI: 0.227**–**0.392), SFG (Beta = 0.308, 95% CI: 0.226**–**0.391), and STG (Beta = 0.316, 95% CI: 0.239–0.393) all retained significant independent effects on infant HC.

### 3.4 Two-step MR mediation analysis among infant HC, cortical SA and adult cEF

Given the bidirectional associations between infant HC and cortical SA observed in the MR analyses, we next examined whether these bidirectional relationships extended to the mediation framework. Specifically, we tested whether cortical SA is consistent with a mediating role in the pathway from infant HC to adult cEF using two-step MR mediation analysis, with Bonferroni correction applied for multiple testing (*P* < 4.55 ×10^−3^, 0.05/11).

We first examined the direction from infant HC to adult cEF via cortical SA. Evidence consistent with a mediating role was observed for total cortical SA (mediation effect = 0.007, 95% CI: 0.003**–**0.010, *P* = 3.00 ×10^−4^, mediation proportion = 26.0%), as well as for several regional SA measures, including rACC (0.005, 95% CI: 0.002**–**0.007, *P* = 5.00 ×10^−4^, 18.0%), cACC (0.006, 95% CI: 0.002–0.010, *P* = 3.20 ×10^−3^, 22.7%), SFG (0.007, 95% CI: 0.003–0.010, *P* = 3.00 ×10^−4^, 25.6%), STG (0.005, 95% CI: 0.002–0.008, *P* = 3.30 ×10^−3^, 19.3%), and BANKSSTS (0.008, 95% CI: 0.003**–**0.012, *P* = 3.00 ×10^−4^, 29.4%). No significant mediation effect was found for pOrb. We next explored the reverse direction, examining whether infant HC is consistent with a mediating role in the pathway from cortical SA to adult cEF. Evidence consistent with this reverse pathway was observed for total cortical SA (0.007, 95% CI: 0.004**–**0.011, *P* = 1.00 ×10^−4^, 26.3%), rACC (0.006, 95% CI: 0.003**–**0.009, *P* = 5.00 ×10^−4^, 32.7%), and STG (0.008, 95% CI: 0.004–0.012, *P* < 1.00 ×10^−4^, 42.3%). Together, these results suggest bidirectional mediation between infant HC and cortical SA in their associations with adult cEF.

### 3.5 Robustness of the MR Findings

To ensure robustness of the above MR findings, we conducted a series of sensitivity analyses (Supplementary Figures S1–S16; Supplementary Tables S13–S16). Cochran’s Q test revealed no evidence of heterogeneity among the IVs (*P* > 0.05). The MR‑Egger intercept test detected no significant horizontal pleiotropy for most analyses (*P* > 0.05), except for the genetically predicted effect of cACC SA on cEF (*P* = 0.044). The MR‑PRESSO global test confirmed the absence of significant outlier SNPs. Visual inspection of leave‑one‑out, forest, scatter, and funnel plots further supported the robustness of the findings (Figures S1–S16). To assess whether our findings were influenced by the choice of instrument selection threshold, we repeated the primary MR analyses using a more stringent threshold of *P* < 1 × 10^−6^ (Tables S14–S16, Figure S17). At this threshold, the number of available instruments was reduced (Table S15), but the effect estimates remained directionally consistent with those obtained using the primary threshold of *P* < 5 × 10^−6^. Specifically, the genetically predicted effect of infant HC on adult cEF remained consistent at the stricter threshold (Beta = 0.031, 95% CI: 0.019**–**0.042, *P* = 3.13 ×10^−7^). For the cortical SA to cEF analyses, the significant effects of total cortical SA, cACC, SFG, pOrb, STG, and BANKSSTS were replicated at *P* < 1 × 10^−6^, with rACC showing a nominally significant effect before Bonferroni correction. For the bidirectional analyses between infant HC and cortical SA, genetic predisposition to larger infant HC increased SA of total brain and all six regional SA measures at the stricter threshold. In the reverse direction, SA of total brain, rACC, and STG showed significant genetically predicted effects on infant HC, while SFG showed a nominally significant effect before Bonferroni correction. Collectively, these sensitivity analyses confirm the robustness of our primary findings and indicate that the observed effects are not driven by the choice of a relaxed instrument selection threshold.

## 4 Discussion

Our findings suggest that genetically driven variation in infant HC is directionally associated with adult cEF, and that cortical SA is consistent with a partial mediating role in this relationship. Together, these results identify early postnatal HC as a developmentally specific biological substrate that shapes lifelong executive capacity.

Our results address a long-standing question in cognitive neuroscience and developmental neurogenetics concerning whether early brain growth merely correlates with adult cognitive function or actively contributes to it. By integrating LDSC and bidirectional MR, we provide convergent evidence that genetic factors influencing infant HC are causally associated with later EF, whereas birth HC does not show such an association. The absence of a significant genetic correlation or genetically predicted effect for birth HC may reflect that neonatal HC is more strongly influenced by obstetric factors and intrauterine constraints, as evidenced by the independent contribution of maternal height to birth HC even in the absence of genetic relatedness (Rice and Thapar, 2010) and by the genetic correlation between maternal birth canal and neonatal head size (Xu et al., 2025). In contrast, postnatal HC during infancy capture active brain expansion during a critical window of synaptogenesis, gliogenesis, and cortical arealization (Ball et al., 2024, Bhaduri et al., 2021, Tsigaras et al., 2026, van Dyck and Morrow, 2017). This developmental specificity is biologically meaningful, as postnatal brain growth is characterized by rapid cortical expansion driven by symmetric division of radial glial progenitors and tangential arealization of the neocortex (Coquand et al., 2024, Liao et al., 2025, Mihailov et al., 2025, Rakic, 2009, Sorrells, 2024),a process captured by infant HC. This distinction is further supported by longitudinal studies showing that postnatal head growth, rather than birth HC, predicts later cognitive outcomes (Horner et al., 2025, Selvanathan et al., 2022). Prior population-based studies have reported associations between childhood head size, intelligence, and educational attainment, but causal directionality has remained unclear due to confounding by socioeconomic and environmental factors (Bann et al., 2023, Feng et al., 2025, Gale et al., 2006, Nicolaou et al., 2020, Thomas and Coecke, 2023). Our MR analyses overcome these limitations and demonstrate that the direction of effect flows from early-life HC measures to adult EF, rather than the reverse.

A central contribution of this work is the identification of cortical SA as the primary neuroanatomical mediator linking infant HC to EF. We observed widespread positive genetic correlations between cEF and total as well as regional cortical SA across frontal, parietal, and temporal association cortices, whereas cortical TH showed no significant associations after correction. This dissociation aligns with evidence that SA and TH are genetically distinct traits shaped by largely independent developmental mechanisms (Grasby et al., 2020, Panizzon et al., 2009, Warrier et al., 2023), with SA expansion reflecting early progenitor proliferation and cortical arealization (Coquand et al., 2024, Liao et al., 2025, Rakic, 2009), and TH more strongly influenced by later synaptic pruning and myelination (Bottenhorn et al., 2025, Whitaker et al., 2016). Our univariable two-sample MR analyses showed that genetically increased SA in multiple executive relevant regions, including the ACC, SFG, pOrb, STG, BANKSSTS, leads to higher cEF performance. Importantly, MVMR analyses confirmed that regional SA effects on cEF remained significant after mutual adjustment for total SA and other regional SA measures, suggesting that both global and regional cortical expansion contribute independently to adult executive function, rather than regional effects being merely a reflection of global brain size or other regional SA. These findings converge with prior work identifying the ACC as a core node for cognitive control (Friedman and Robbins, 2022, Klein-Flügge et al., 2022), conflict monitoring (Cipolotti et al., 2025), and effort allocation (Clairis and Lopez-Persem, 2023, Clairis and Pessiglione, 2024), and the lateral and orbital prefrontal cortices as essential substrates for working memory (Hallenbeck et al., 2025), planning (Friedman and Robbins, 2022), and decision-making (Klein-Flügge et al., 2022, Shi et al., 2023). Temporal association regions, including the STG and BANKSSTS, support higher-order multimodal integration and social-cognitive processes increasingly recognized as integral components of executive control networks (Assem et al., 2024, Beauchamp, 2015, Deen et al., 2015). Importantly, reverse genetically predicted effects from EF to cortical SA were not observed, reinforcing a neurodevelopment to cognition directionality.

Having established cortical SA as a key mediator, we next characterize its developmental relationship with infant HC. Our findings reveal a complex bidirectional relationship. Bidirectional MR showed significant effects in both directions, and MVMR further demonstrated that the reverse effects from cortical SA to infant HC were not merely explained by genetic overlap among regions, as total SA and regional SA measures retained significant independent effects on infant HC. This suggests that both global and regional cortical expansion may actively contribute to early HC, consistent with the view that cortical development and early cranial growth are mutually reinforcing during infancy (Ball et al., 2024, Bonthrone et al., 2025, Davis et al., 2025, Natu et al., 2021, Walhovd et al., 2024). This bidirectional interplay highlights the dynamic nature of early brain development, in which cortical structure and overall brain size may shape each other during critical developmental windows.

Two-step MR mediation analyses further integrated these findings, revealing bidirectional mediation between infant HC and cortical SA in their associations with adult cEF. Cortical SA partially mediated the pathway from infant HC to adult cEF, while the reverse mediation suggested that the association between cortical SA and cEF is partially explained by early brain size: individuals with genetic predisposition for larger cortical SA also tend to have larger infant HC, which in turn supports better executive function. Together, these bidirectional mediation findings indicate that infant HC and cortical SA are developmentally intertwined, each partially accounting for the other’s association with adult executive function. The remaining direct effect suggests additional pathways, such as subcortical development (Alex et al., 2024, Chen et al., 2023), white matter connectivity (Carozza et al., 2025, Goddings et al., 2021), or early network organization (Baum et al., 2020, Yin et al., 2025), that are not captured by cortical SA alone.

These results help reconcile inconsistencies in the literature linking brain size and cognitive ability. While total brain volume shows only weak associations with cognition (Pietschnig et al., 2015), our findings highlight cortical SA as the critical neuroanatomical dimension connecting early development to EF. This aligns with evidence that SA, but not thickness, is genetically correlated with general cognitive ability and educational attainment (Grasby et al., 2020, Grotzinger et al., 2023, Huang et al., 2022, Makowski et al., 2023), whereas global brain volume conflates two developmentally distinct traits with differential relationships to cognition (Moodie et al., 2025). By leveraging genetically anchored variation and causal inference, our study provides evidence that early cortical expansion may constitute a biologically meaningful pathway to adult executive differences.

Several limitations should be acknowledged. First, HC is only an indirect proxy for early brain size, influenced by skull morphology, somatic growth, nutrition, and other developmental factors (Haworth et al., 2019, Taal et al., 2012, the Early Growth Genetics, C, 2022), and cannot capture finer-grained neuroanatomical features such as gyrification, white matter maturation, or subcortical growth. Thus, our MR analyses primarily assess genetic liability associated with HC rather than brain growth itself, and this limitation should be considered when interpreting our findings. Second, our mediation pathway is subject to an important temporal limitation. HC was measured in infancy, whereas cortical SA and cEF were measured in adulthood and not in the same individuals. Therefore, our results should be interpreted as evidence for a genetically informed indirect pathway consistent with a developmental interpretation, rather than as direct chronological mediation. An alternative interpretation is that genetic liability for early head growth may independently influence both adult cortical structure and cognition via shared developmental genetic mechanisms (Korologou-Linden et al., 2023).Third, the cEF phenotype was derived from UK Biobank cognitive tasks that were not originally designed as canonical executive-function paradigms. Although this factor has been validated as capturing shared executive variance across tasks (Hatoum et al., 2023), it may not fully capture the full breadth of executive functions. Future studies using tasks specifically targeting inhibition, updating, and shifting would help validate and extend our findings. Fourth, all GWAS summary statistics were derived from individuals of European ancestry, which may limit generalizability to other populations. Fifth, although MR mitigates confounding, it relies on the validity of genetic instruments and cannot completely exclude subtle pleiotropic effects (Burgess and Cronjé, 2024, Hu et al., 2024, Lee and Lee, 2025, Sanderson et al., 2021, Verbanck et al., 2018). Sixth, although MVMR analyses supported independent effects of regional SA on cEF and infant HC, the conditional F-statistics were below the conventional threshold of 10, indicating weak instrument strength under the conventional IVW framework. We therefore relied on the dIVW estimator, which has been shown to provide reliable estimates under weak instruments when condition statistics exceed 20(Wu et al., 2025). Nevertheless, these MVMR results should be interpreted with caution, and replication in studies with stronger instruments is warranted. Seventh, despite careful GWAS selection, a small sample overlap remained between HC and cortical structure datasets (n = 391), which may bias estimates toward the null. Eighth, although most MR analyses passed pleiotropy tests, the genetically predicted effect of cACC SA on cEF showed a nominally significant MR-Egger intercept (*P* = 0.044), indicating possible directional pleiotropy. This particular finding should therefore be interpreted with caution, and replication in independent samples is warranted.

Future studies with longitudinal designs spanning infancy to adulthood, incorporating multimodal neuroimaging and environmental measures, are needed to further characterize the developmental trajectories connecting early brain size to adult cognitive outcomes. In particular, examining gene‑by‑environment interactions, including nutrition, socioeconomic status, and early‑life stress, could provide insights into factors that moderate these developmental pathways.

In conclusion, our findings provide genetic evidence consistent with a pathway from early-life HC to adult executive function, primarily through cortical SA expansion, with bidirectional mediation between infant HC and cortical SA. By linking infant neurodevelopment, cortical morphology, and higher-order cognition within a unified genetically informed framework, this work advances a lifespan perspective on executive function and highlights early postnatal development as a critical window for the biological foundations of cognitive control.

## CRediT authorship contribution statement

**Qingjing Jia:** Writing – original draft, Visualization, Validation, Methodology, Formal analysis, Data curation. **Dekang Wang:** Visualization, Validation. **Junjiao Feng:** Writing – review & editing, Writing – original draft, Visualization, Validation, Supervision, Software, Methodology, Investigation, Funding acquisition, Formal analysis, Data curation, Conceptualization. **Feng Liu:** Writing – review & editing, Visualization, Methodology, Conceptualization.

## Funding

This work was supported by the 10.13039/501100001809National Natural Science Foundation of China (Grant number 32300875) awarded to Junjiao Feng.

## Declaration of Competing Interest

Ethical approval and consent to participate: This MR study utilized de-identified summary-level data that are publicly accessible. Informed consent and ethical approval were obtained for all original GWAS studies, and therefore, no further ethical approval was necessary for this study. All authors approve the publication of the manuscript in its current form. All authors declare no conflicts of interest.

## Data Availability

I have shared the link to my data/code at the Attach file step
