# Supplementary material for: Early‑life head circumference is causally associated with adult executive function through cortical surface area expansion
Source: Dev Cogn Neurosci. 2026 Aug 29;81:101805. doi: 10.1016/j.dcn.2026.101805 (PMC13571153; doi:10.1016/j.dcn.2026.101805)
Supplement: Supplementary file 1 [file mmc1.docx]

| **Supplementary tables** | |
| --- | --- |
| **Table** | **Contents** |
| Table S1 | Cohort descriptions for the GWAS meta-analysis of early-life HC |
| Table S2 | Cohort descriptions for the GWAS meta-analysis of cortical structural phenotypes |
| Table S3 | Genetic correlations between early-life HC and adult cEF |
| Table S4 | Bidirectional MR estimates between infant HC and adult cEF |
| Table S5 | Genetic correlations between cortical structural phenotypes and adult cEF |
| Table S6 | Bidirectional MR estimates between cortical SA and adult cEF |
| Table S7 | MVMR analyses showed genetically predicted effects of cortical SA on adult cEF |
| Table S8 | Genetic correlations between infant HC and cortical SA |
| Table S9 | Bidirectional MR estimates between infant HC and cortical SA |
| Table S10 | MVMR analyses showed genetically predicted effects of cortical SA on infant HC |
| Table S11 | Mediation analyses among infant HC, cortical SA and adult cEF |
| Table S12 | IVs used for each exposure-outcome pairs in the MR analyses |
| Table S13 | Sensitivity analysis results for all exposure-outcome pairs in MR analyses |
| Table S14 | Sensitivity analysis of primary MR estimates using a more stringent instrument selection threshold (*P* < 1e-6) |
| Table S15 | IVs for sensitivity analyses using a more stringent instrument selection threshold (*P* < 1e-6) |
| Table S16 | Sensitivity analysis results (heterogeneity and pleiotropy tests) using a more stringent instrument selection threshold (*P* < 1e-6) |

| **Table S1. Cohort descriptions for the GWAS meta-analysis of early-life HC** | | | | | | | | | | |
| --- | --- | --- | --- | --- | --- | --- | --- | --- | --- | --- |
|  |  |  | **Birth HC** | | | | **Infant HC** | | | |
| **Study name** | **HC** | **Ethnicity** | **Sample size** | **Mean Birth HC (SD), cm** | **% males in analysis** | **Median gestational age (90% range), wks** | **Sample size** | **Mean Infant HC (SD), cm** | **% males in analysis** | **Mean age (SD) at HC measurement, months** |
| **ABCD** | Infant | Caucasian (Dutch) | NA | NA | NA | NA | 1018 | 45.6 (1.7) | 48.4 | 10.0 (2.5) |
| **ALSPAC** | Birth and Infant | European | 5690 | 35.0 (1.4) | 51 | 40.0 (37.0-42.0) | 3960 | 48.6(1.6) | 51.5 | 20.2 (2.7) |
| **CHOP (Philadelphia)** | Birth and Infant | European | 300 | 34.8 (1.5) | 58.7 | 39.0 (38.0-41.0) | 856 | 47.6 (1.9) | 60.4 | 18.4 (3.5) |
| **COPSAC 2000** | Birth and Infant | Danish (Caucasian) | 345 | 35.2 (1.5) | 49.2 | 40.0 (39.0-41.0) | 325 | 46.7 (1.5) | 49.1 | 12.5 (0.8) |
| **COPSAC 2010** | Birth and Infant | Danish (Caucasian) | 594 | 35.1 (1.6) | 51.1 | 40.1 (39.1-41) | 603 | 46.5 (1.5) | 51.1 | 12.3 (0.5) |
| **COPSAC registry** | Birth | Danish (Caucasian) | 441 | 35.4 (1.5) | 68.7 | 40.0 (39.0-41.0) | NA | NA | NA | NA |
| **DNBC-PTB** | Birth and Infant | Danish (Caucasian) | 989 | 35.5 (1.5) | 52.7 | 40.4 (39.6-40.9) | 508 | 46.2 (1.7) | 53.5 | 11.3 (2.4) |
| **Generation R** | Birth and Infant | European | 1859 | 34.1 (1.6) | 51.7 | 40.4 (38.0; 42.0) | 2299 | 46.5 (1.6) | 51.1 | 12.4 (2.5) |
| **GINILISA** | Birth and Infant | Caucasian (German) | 1350 | 34.9 (1.3) | 51.6 | 39.8 (38.0; 42.0) | 1455 | 47.6 (2.0) | 52.1 | 17.7 (6.0) |
| **DNBC GOYA-offspring** | Birth and Infant | Danish (Caucasian) | 895 | 35.6 (1.6) | 51 | 40.2 (38.4-42.0) | 235 | 44 | 13.4 | 13.4 (0.7) |
| **HAPO** | Birth | European | 1363 | 34.9 (1.5) | 49.7 | 40.0 (39.9; 40.1) | NA | NA | NA | NA |
| **INMA MEN, SAB, VAL** | Birth and Infant | European | 942 | 34.2 (1.5) | 52.1 | 39.9 (38.0-41.3) | 550 | 47.8 (1.5) | 51.8 | 17.6 (2.2) |
| **LIFE-Child** | Birth and Infant | European | 624 | 35.0 (1.4) | 52.1 | 40.0 (37.0-41.0) | 1365 | 47.7 (2.0) | 50.7 | 19.2 (5.5) |
| **MoBa (Bo Jacobsson)** | Birth and Infant | Norwegian | 579 | 35.4 (1.21) | 46.5 | 40.0 (39.1-40.9) | 839 | 46.1 (2.1) | 49.3 | 11.0 (2.4) |
| **MoBa (Pål R. Njølstad)** | Birth and Infant | Norwegian | 9193 | 35.5 (1.4) | 51.8 | 40.3 (37.6- 42.1) | 5644 | 46.9 (1.4) | 51.7 | 12.2 (1.5) |
| **NFBC1966** | Infant | Finnish | NA | NA | NA | NA | 4603 | 46.8 (1.5) | 47.8 | 12.3 (0.9) |
| **NFBC1986** | Birth and Infant | Finnish | 1398 | 35.1 (1.6) | 46.1 | 40 (39 - 41) | 826 | 46.7 (1.6) | 44.4 | 12.3 (0.8) |
| **PANIC** | Birth and Infant | Caucasian | 452 | 35.0(1.6) | 52.2 | 41.0(38.0-42.0) | 370 | 46.8(1.6) | 51.9 | 11.7 (1.4) |
| **Raine Study** | Birth and Infant | Caucasian (Australian) | 1344 | 34.7 (1.5) | 52 | 39.86 (37.6,41.9 | 1373 | 47.5 (1.4) | 51.5 | 13.9 (1.2) |
| **SKOT 1** | Birth and Infant | Danish (Caucasian) | 167 | 35.1 (1.3) | 46 | 40.2 (38.3-41.9) | 247 | 47.9 (1.5) | 48.0 | 18.0 (0.6) |
| **SKOT 2** | Birth and Infant | Danish (Caucasian) | 112 | 35.3 (1.6) | 55 | 40.0 (38.3-41.7) | 104 | 48.5 (1.7) | 56.0 | 18.1 (0.4) |
| **STRIP** | Birth and Infant | Finnish | 584 | 35.0 (1.5) | 53.4 | 39.0 (37.2-41.0) | 833 | 47.3 (1.4) | 51.0 | 13.1 (0.3) |
| **TDCOB** | Birth | Danish (Caucasian) | 1208 | 35.0 (1.9) | 44 | 39.7 (37.0-42.0) | NA | NA | NA | NA |
| **TEENAGE** | Birth and Infant | Greek | 197 | 34.6 (1.4) | 46 | 40.0 (2) | 327 | 47.1 (1.7) | 43.0 | 14.7 (3.9) |

| **Table S2. Cohort descriptions for the GWAS meta-analysis of cortical structural phenotypes** | | | | |
| --- | --- | --- | --- | --- |
| **Cohort** | **Total N** | **Females** | **Mean Age** | **Healthy N** |
| 1000BRAINS | 775 | 346 | 67.3 | 775 |
| ADNI1 | 735 | 299 | 74.8 | 204 |
| ADNI2GO | 649 | 297 | 72.4 | 564 |
| ALSPAC | 391 | 0 | 19.6 | 391 |
| ASRB | 233 | 138 | 38.5 | 75 |
| BETULA | 311 | 169 | 62.4 | 311 |
| BIG-Affy | 1180 | 688 | 22.6 | 1180 |
| BIG-PsychChip | 432 | 206 | 22.5 | 432 |
| BONN | 102 | 0 | 38.2 | 102 |
| BrainScale | 242 | 131 | 10.0 | 242 |
| CARDIFF | 270 | 194 | 24.8 | 270 |
| DNS-V3 | 324 | 168 | 19.7 | 324 |
| DNS-V4 | 191 | 108 | 19.9 | 191 |
| EPIGEN | 178 | 104 | 38.4 | 0 |
| FOR2107 | 785 | 474 | 34.4 | 416 |
| GIG | 283 | 168 | 24.2 | 283 |
| GSP | 442 | 251 | 21.4 | 442 |
| HUBIN | 177 | 55 | 41.9 | 97 |
| HUNT | 876 | 462 | 58.9 | 876 |
| IMAGEN | 1358 | 725 | 14.6 | 1358 |
| IMpACT | 238 | 140 | 40.8 | 113 |
| LBC1936 | 604 | 285 | 72.7 | 604 |
| LIBD | 484 | 214 | 33.2 | 310 |
| MCIC | 162 | 56 | 33.7 | 94 |
| MooDS | 282 | 129 | 33.6 | 282 |
| MPIP | 550 | 318 | 48.3 | 177 |
| MPRC | 387 | 205 | 37.2 | 214 |
| MÜNSTER | 985 | 561 | 35.8 | 741 |
| NCNG | 321 | 218 | 51.6 | 321 |
| NESDA | 254 | 171 | 37.5 | 55 |
| NeuroIMAGE | 210 | 68 | 17.1 | 61 |
| NTR | 322 | 197 | 29.4 | 322 |
| OATS | 360 | 237 | 70.5 | 360 |
| PAFIP | 112 | 42 | 28.3 | 14 |
| PDNZ | 164 | 56 | 68.2 | 47 |
| PING | 337 | 151 | 11.8 | 337 |
| PPMI | 414 | 137 | 61.7 | 124 |
| QTIM | 996 | 645 | 22.4 | 996 |
| SHIP | 1118 | 579 | 55.8 | 1118 |
| SHIP-Trend | 891 | 499 | 50.4 | 891 |
| Sydney MAS | 494 | 274 | 78.4 | 494 |
| SYS | 1675 | 884 | 28.3 | 1675 |
| TCD-NUIG | 192 | 108 | 29.9 | 156 |
| TOP | 505 | 253 | 35.2 | 216 |
| TOP3T | 400 | 181 | 33.2 | 296 |
| UiO2016 | 229 | 98 | 31.8 | 90 |
| UiO2017 | 308 | 173 | 42.1 | 247 |
| UMCU | 698 | 373 | 33.1 | 548 |

| **Table S3. Genetic correlations between early-life HC and adult cEF** | | | | | |
| --- | --- | --- | --- | --- | --- |
| **Phenotype1** | **Phenotype2** | **Genetic correlation (R_g_)** | **SE** | **Z** | ***P-*value** |
| cEF | birth HC | 0.128 | 0.076 | 1.689 | 0.091 |
| cEF | infant HC | 0.196 | 0.047 | 4.173 | 3.01E-05 |

| **Table S4. Bidirectional MR estimates between infant HC and adult cEF** | | | | | | | | | | | | | | | | | | | | | |
| --- | --- | --- | --- | --- | --- | --- | --- | --- | --- | --- | --- | --- | --- | --- | --- | --- | --- | --- | --- | --- | --- |
| Note: Causal effects estimated using 5 two-sample Mendelian randomization methods. All statistical tests were two-sided. *P*-value <2.50e-2 after Bonferroni correction was considered significant. | | | | | | | | | | | | | | | | | | | | | |
| **Exposure** | **Outcome** | **Inverse-variance weighted** | | | | **MR-Egger** | | | | **Weighted median** | | | | **Weighted mode** | | | | **MR RAPS** | | | |
|  |  | **Estimate (β)** | **95% lower CI** | **95% upper CI** | ***P-*value** | **Estimate (β)** | **95% lower CI** | **95% upper CI** | ***P-*value** | **Estimate (β)** | **95% lower CI** | **95% upper CI** | ***P-*value** | **Estimate (β)** | **95% lower CI** | **95% upper CI** | ***P-*value** | **Estimate (β)** | **95% lower CI** | **95% upper CI** | ***P-*value** |
| infant HC | cEF | 0.026 | 0.016 | 0.035 | 3.83E-07 | 0.035 | -0.003 | 0.073 | 9.04E-02 | 0.020 | 0.006 | 0.034 | 6.12E-03 | 0.013 | -0.015 | 0.041 | 3.76E-01 | 0.027 | 0.016 | 0.037 | 3.55E-07 |
| cEF | infant HC | -0.216 | -0.602 | 0.170 | 2.72E-01 | 0.308 | -1.619 | 2.235 | 7.55E-01 | -0.417 | -0.963 | 0.129 | 1.35E-01 | -0.467 | -1.624 | 0.690 | 4.32E-01 | -0.223 | -0.598 | 0.153 | 2.46E-01 |

| **Table S5. Genetic correlations between cortical structural phenotypes and adult cEF** | | | | | | | |
| --- | --- | --- | --- | --- | --- | --- | --- |
| **Phenotype1** | | | **Phenotype2** | ***Genetic correlation*** | **SE** | ***Z*** | ***P-*value** |
|  |  |  |  |  |  |  |  |
| Total brain | surface area | | cEF | 0.157 | 0.036 | 4.321 | 1.55E-05 |
|  | Thickness | | cEF | 0.030 | 0.043 | 0.694 | 4.88E-01 |
| Frontal | surface area | Frontal Pole | cEF | 0.091 | 0.059 | 1.541 | 1.23E-01 |
|  |  | Medial Orbitofrontal | cEF | 0.213 | 0.043 | 5.007 | 5.53E-07 |
|  |  | Lateral Orbitofrontal | cEF | 0.137 | 0.042 | 3.305 | 1.00E-03 |
|  |  | Rostral Anterior Cingulate | cEF | 0.179 | 0.041 | 4.316 | 1.59E-05 |
|  |  | Caudal Anterior Cingulate | cEF | 0.226 | 0.048 | 4.724 | 2.31E-06 |
|  |  | Superior Frontal | cEF | 0.141 | 0.040 | 3.491 | 5.00E-04 |
|  |  | Rostral Middle Frontal | cEF | 0.125 | 0.040 | 3.145 | 1.70E-03 |
|  |  | Pars Orbitalis | cEF | 0.176 | 0.046 | 3.827 | 1.00E-04 |
|  |  | Pars Triangularis | cEF | 0.115 | 0.046 | 2.513 | 1.20E-02 |
|  |  | Pars Opercularis | cEF | 0.066 | 0.053 | 1.234 | 2.17E-01 |
|  |  | Caudal Middle Frontal | cEF | 0.079 | 0.045 | 1.769 | 7.68E-02 |
|  |  | Paracentral | cEF | 0.127 | 0.046 | 2.766 | 5.70E-03 |
|  |  | Precentral | cEF | 0.112 | 0.040 | 2.809 | 5.00E-03 |
|  | Thickness | Frontal Pole | cEF | -0.026 | 0.129 | -0.200 | 8.41E-01 |
|  |  | Medial Orbitofrontal | cEF | -0.062 | 0.063 | -0.986 | 3.24E-01 |
|  |  | Lateral Orbitofrontal | cEF | -0.017 | 0.061 | -0.276 | 7.82E-01 |
|  |  | Rostral Anterior Cingulate | cEF | -0.113 | 0.062 | -1.818 | 6.90E-02 |
|  |  | Caudal Anterior Cingulate | cEF | -0.009 | 0.050 | -0.176 | 8.60E-01 |
|  |  | Superior Frontal | cEF | -0.004 | 0.047 | -0.094 | 9.25E-01 |
|  |  | Rostral Middle Frontal | cEF | 0.048 | 0.048 | 0.993 | 3.21E-01 |
|  |  | Pars Orbitalis | cEF | -0.043 | 0.056 | -0.769 | 4.42E-01 |
|  |  | Pars Triangularis | cEF | -0.015 | 0.048 | -0.316 | 7.52E-01 |
|  |  | Pars Opercularis | cEF | 0.053 | 0.047 | 1.129 | 2.59E-01 |
|  |  | Caudal Middle Frontal | cEF | 0.047 | 0.047 | 0.987 | 3.24E-01 |
|  |  | Paracentral | cEF | 0.042 | 0.043 | 0.958 | 3.38E-01 |
|  |  | Precentral | cEF | 0.149 | 0.050 | 2.963 | 3.00E-03 |
| Pareital | surface area | Postcentral | cEF | 0.099 | 0.042 | 2.376 | 1.75E-02 |
|  |  | Precuneus | cEF | 0.200 | 0.041 | 4.831 | 1.36E-06 |
|  |  | Superior Parietal | cEF | 0.154 | 0.041 | 3.726 | 2.00E-04 |
|  |  | Supramarginal | cEF | 0.138 | 0.047 | 2.919 | 3.50E-03 |
|  |  | Inferior Parietal | cEF | 0.112 | 0.041 | 2.746 | 6.00E-03 |
|  |  | Posterior Cingulate | cEF | 0.179 | 0.042 | 4.319 | 1.57E-05 |
|  |  | Isthmus Cingulate | cEF | 0.094 | 0.042 | 2.214 | 2.68E-02 |
|  | Thickness | Postcentral | cEF | 0.100 | 0.052 | 1.921 | 5.47E-02 |
|  |  | Precuneus | cEF | 0.005 | 0.047 | 0.106 | 9.16E-01 |
|  |  | Superior Parietal | cEF | 0.057 | 0.044 | 1.287 | 1.98E-01 |
|  |  | Supramarginal | cEF | 0.000 | 0.043 | -0.009 | 9.93E-01 |
|  |  | Inferior Parietal | cEF | -0.025 | 0.044 | -0.572 | 5.68E-01 |
|  |  | Posterior Cingulate | cEF | 0.030 | 0.052 | 0.585 | 5.58E-01 |
|  |  | Isthmus Cingulate | cEF | 0.028 | 0.048 | 0.589 | 5.56E-01 |
| Temporal | surface area | Insula | cEF | 0.090 | 0.037 | 2.432 | 1.50E-02 |
|  |  | Entorhinal | cEF | 0.202 | 0.049 | 4.111 | 3.94E-05 |
|  |  | Parahippocampal | cEF | 0.134 | 0.049 | 2.738 | 6.20E-03 |
|  |  | Fusiform | cEF | 0.134 | 0.042 | 3.202 | 1.40E-03 |
|  |  | Temporal Pole | cEF | 0.071 | 0.046 | 1.547 | 1.22E-01 |
|  |  | Inferior Temporal | cEF | 0.113 | 0.042 | 2.669 | 7.60E-03 |
|  |  | Middle Temporal | cEF | 0.095 | 0.038 | 2.486 | 1.29E-02 |
|  |  | Superior Temporal | cEF | 0.150 | 0.036 | 4.182 | 2.89E-05 |
|  |  | Banks of the Superior Temporal Sulcus | cEF | 0.153 | 0.044 | 3.484 | 5.00E-04 |
|  |  | Transverse Temporal | cEF | 0.088 | 0.037 | 2.386 | 1.70E-02 |
|  | Thickness | Insula | cEF | -0.011 | 0.049 | -0.231 | 8.17E-01 |
|  |  | Entorhinal | cEF | 0.009 | 0.067 | 0.127 | 8.99E-01 |
|  |  | Parahippocampal | cEF | -0.065 | 0.053 | -1.216 | 2.24E-01 |
|  |  | Fusiform | cEF | -0.041 | 0.056 | -0.741 | 4.59E-01 |
|  |  | Temporal Pole | cEF | -0.035 | 0.078 | -0.450 | 6.53E-01 |
|  |  | Inferior Temporal | cEF | -0.014 | 0.057 | -0.248 | 8.04E-01 |
|  |  | Middle Temporal | cEF | -0.001 | 0.054 | -0.010 | 9.92E-01 |
|  |  | Superior Temporal | cEF | 0.056 | 0.051 | 1.106 | 2.69E-01 |
|  |  | Banks of the Superior Temporal Sulcus | cEF | -0.014 | 0.060 | -0.226 | 8.22E-01 |
|  |  | Transverse Temporal | cEF | 0.102 | 0.055 | 1.849 | 6.44E-02 |
| Occipital | surface area | Lingual | cEF | 0.038 | 0.041 | 0.915 | 3.60E-01 |
|  |  | Pericalcarine | cEF | 0.015 | 0.039 | 0.378 | 7.06E-01 |
|  |  | Cuneus | cEF | 0.035 | 0.040 | 0.857 | 3.92E-01 |
|  |  | Lateral Occipital | cEF | 0.062 | 0.035 | 1.770 | 7.68E-02 |
|  | Thickness | Lingual | cEF | 0.084 | 0.052 | 1.598 | 1.10E-01 |
|  |  | Pericalcarine | cEF | 0.089 | 0.070 | 1.262 | 2.07E-01 |
|  |  | Cuneus | cEF | 0.093 | 0.069 | 1.351 | 1.77E-01 |
|  |  | Lateral Occipital | cEF | 0.027 | 0.057 | 0.473 | 6.37E-01 |

| **Table S6. Bidirectional MR estimates between cortical SA and adult cEF** | | | | | | | | | | | | | | | | | | | | | | | | | |
| --- | --- | --- | --- | --- | --- | --- | --- | --- | --- | --- | --- | --- | --- | --- | --- | --- | --- | --- | --- | --- | --- | --- | --- | --- | --- |
| Note: Causal effects estimated using 5 two-sample Mendelian randomization methods. All statistical tests were two-sided. *P*-value < 2.08e-3 after Bonferroni correction was considered significant. | | | | | | | | | | | | | | | | | | | | | | | | | |
| **Exposure** | | | **Outcome** | | | **Inverse-variance weighted** | | | | **MR-Egger** | | | | **Weighted median** | | | | **Weighted mode** | | | | **MR RAPS** | | | |
|  |  |  |  |  |  | **Estimate (β)** | **95% lower CI** | **95% upper CI** | ***P-*value** | **Estimate (β)** | **95% lower CI** | **95% upper CI** | ***P-*value** | **Estimate (β)** | **95% lower CI** | **95% upper CI** | ***P-*value** | **Estimate (β)** | **95% lower CI** | **95% upper CI** | ***P-*value** | **Estimate (β)** | **95% lower CI** | **95% upper CI** | ***P-*value** |
| Total brain | surface area |  | cEF | | | 0.028 | 0.016 | 0.040 | 2.97E-06 | 0.016 | -0.024 | 0.057 | 4.37E-01 | 0.020 | 0.003 | 0.036 | 1.86E-02 | 0.017 | -0.014 | 0.048 | 2.90E-01 | 0.029 | 0.018 | 0.040 | 5.14E-07 |
| Frontal | surface area | Medial Orbitofrontal |  |  |  | 0.013 | 0.001 | 0.024 | 2.66E-02 | 0.009 | -0.026 | 0.043 | 6.26E-01 | 0.014 | -0.001 | 0.030 | 6.39E-02 | 0.032 | -0.001 | 0.066 | 7.32E-02 | 0.013 | 0.003 | 0.024 | 1.28E-02 |
|  |  | Rostral Anterior Cingulate |  |  |  | 0.018 | 0.009 | 0.027 | 5.90E-05 | 0.013 | -0.020 | 0.045 | 4.52E-01 | 0.016 | 0.002 | 0.029 | 2.21E-02 | 0.004 | -0.023 | 0.032 | 7.49E-01 | 0.019 | 0.009 | 0.029 | 3.54E-04 |
|  |  | Caudal Anterior Cingulate |  |  |  | 0.031 | 0.016 | 0.046 | 4.96E-05 | -0.001 | -0.033 | 0.030 | 9.29E-01 | 0.019 | 0.001 | 0.038 | 3.95E-02 | 0.010 | -0.019 | 0.040 | 5.13E-01 | 0.033 | 0.020 | 0.046 | 2.71E-07 |
|  |  | Superior Frontal |  |  |  | 0.026 | 0.014 | 0.039 | 3.30E-05 | 0.031 | -0.010 | 0.073 | 1.61E-01 | 0.027 | 0.008 | 0.046 | 5.46E-03 | 0.029 | -0.005 | 0.062 | 1.14E-01 | 0.027 | 0.013 | 0.042 | 2.64E-04 |
|  |  | Pars Orbitalis |  |  |  | 0.029 | 0.016 | 0.042 | 1.09E-05 | 0.029 | -0.019 | 0.077 | 2.52E-01 | 0.022 | 0.005 | 0.040 | 1.31E-02 | 0.006 | -0.027 | 0.039 | 7.32E-01 | 0.030 | 0.018 | 0.043 | 2.48E-06 |
| Parietal | surface area | Precuneus |  |  |  | 0.001 | -0.011 | 0.013 | 8.66E-01 | -0.016 | -0.049 | 0.017 | 3.44E-01 | 0.001 | -0.015 | 0.018 | 8.60E-01 | 0.008 | -0.021 | 0.037 | 5.89E-01 | 0.001 | -0.010 | 0.012 | 8.46E-01 |
|  |  | Superior Parietal |  |  |  | 0.012 | 0.002 | 0.023 | 1.97E-02 | -0.014 | -0.045 | 0.016 | 3.64E-01 | 0.011 | -0.002 | 0.025 | 1.06E-01 | 0.011 | -0.013 | 0.036 | 3.81E-01 | 0.013 | 0.003 | 0.023 | 9.27E-03 |
|  |  | Posterior Cingulate |  |  |  | 0.001 | -0.011 | 0.013 | 8.63E-01 | 0.008 | -0.034 | 0.050 | 7.07E-01 | -0.011 | -0.028 | 0.007 | 2.31E-01 | -0.016 | -0.050 | 0.018 | 3.73E-01 | 0.001 | -0.012 | 0.014 | 8.67E-01 |
| Temporal | surface area | Entorhinal |  |  |  | 0.009 | -0.003 | 0.021 | 1.31E-01 | -0.013 | -0.047 | 0.021 | 4.62E-01 | 0.003 | -0.014 | 0.020 | 7.50E-01 | -0.006 | -0.034 | 0.023 | 7.07E-01 | 0.010 | -0.003 | 0.022 | 1.31E-01 |
|  |  | Superior Temporal |  |  |  | 0.019 | 0.008 | 0.030 | 1.13E-03 | 0.003 | -0.049 | 0.055 | 9.11E-01 | 0.018 | 0.002 | 0.033 | 2.55E-02 | 0.014 | -0.024 | 0.051 | 4.86E-01 | 0.020 | 0.009 | 0.031 | 2.18E-04 |
|  |  | Banks of the Superior Temporal Sulcus |  |  |  | 0.033 | 0.020 | 0.046 | 8.32E-07 | 0.009 | -0.029 | 0.047 | 6.48E-01 | 0.036 | 0.019 | 0.053 | 3.94E-05 | 0.041 | 0.008 | 0.075 | 2.88E-02 | 0.034 | 0.021 | 0.048 | 7.00E-07 |
| cEF | | | Total brain | surface area |  | 0.063 | -0.225 | 0.351 | 6.68E-01 | 0.150 | -1.324 | 1.624 | 8.42E-01 | 0.181 | -0.222 | 0.585 | 3.79E-01 | 0.591 | -0.244 | 1.426 | 1.71E-01 | 0.065 | -0.225 | 0.354 | 6.61E-01 |
|  |  |  | Frontal | surface area | Medial Orbitofrontal | -0.327 | -0.661 | 0.008 | 5.56E-02 | -0.140 | -1.833 | 1.554 | 8.72E-01 | -0.016 | -0.466 | 0.435 | 9.46E-01 | 0.393 | -0.580 | 1.366 | 4.32E-01 | -0.336 | -0.659 | -0.013 | 4.13E-02 |
|  |  |  |  |  | Rostral Anterior Cingulate | 0.105 | -0.241 | 0.450 | 5.52E-01 | 0.254 | -1.509 | 2.016 | 7.79E-01 | 0.211 | -0.262 | 0.684 | 3.82E-01 | 0.686 | -0.535 | 1.908 | 2.75E-01 | 0.108 | -0.221 | 0.436 | 5.20E-01 |
|  |  |  |  |  | Caudal Anterior Cingulate | 0.032 | -0.319 | 0.382 | 8.59E-01 | -0.809 | -2.589 | 0.970 | 3.76E-01 | 0.067 | -0.454 | 0.587 | 8.02E-01 | -0.089 | -1.269 | 1.091 | 8.83E-01 | 0.033 | -0.307 | 0.373 | 8.50E-01 |
|  |  |  |  |  | Superior Frontal | -0.131 | -0.435 | 0.174 | 4.00E-01 | 0.777 | -0.762 | 2.315 | 3.26E-01 | -0.015 | -0.445 | 0.415 | 9.44E-01 | 0.099 | -0.833 | 1.031 | 8.36E-01 | -0.134 | -0.439 | 0.171 | 3.88E-01 |
|  |  |  |  |  | Pars Orbitalis | -0.040 | -0.385 | 0.305 | 8.19E-01 | 0.805 | -0.944 | 2.554 | 3.70E-01 | 0.093 | -0.374 | 0.560 | 6.96E-01 | 0.628 | -0.383 | 1.639 | 2.28E-01 | -0.042 | -0.354 | 0.271 | 7.95E-01 |
|  |  |  | Parietal | surface area | Precuneus | 0.159 | -0.168 | 0.487 | 3.41E-01 | 1.318 | -0.425 | 3.060 | 1.44E-01 | 0.166 | -0.279 | 0.611 | 4.64E-01 | -0.125 | -1.050 | 0.799 | 7.91E-01 | 0.164 | -0.151 | 0.478 | 3.08E-01 |
|  |  |  |  |  | Superior Parietal | 0.288 | -0.044 | 0.620 | 8.91E-02 | 0.259 | -1.472 | 1.990 | 7.70E-01 | 0.369 | -0.086 | 0.824 | 1.12E-01 | 0.582 | -0.316 | 1.480 | 2.09E-01 | 0.296 | -0.024 | 0.616 | 6.96E-02 |
|  |  |  |  |  | Posterior Cingulate | -0.044 | -0.407 | 0.318 | 8.11E-01 | -0.532 | -2.370 | 1.306 | 5.73E-01 | -0.171 | -0.670 | 0.329 | 5.03E-01 | -0.478 | -1.663 | 0.706 | 4.32E-01 | -0.046 | -0.369 | 0.278 | 7.82E-01 |
|  |  |  | Temporal | surface area | Entorhinal | -0.159 | -0.532 | 0.214 | 4.03E-01 | 0.589 | -1.319 | 2.497 | 5.48E-01 | -0.037 | -0.553 | 0.478 | 8.87E-01 | 0.144 | -0.867 | 1.155 | 7.81E-01 | -0.164 | -0.508 | 0.179 | 3.49E-01 |
|  |  |  |  |  | Superior Temporal | 0.085 | -0.222 | 0.391 | 5.89E-01 | 0.074 | -1.534 | 1.682 | 9.29E-01 | 0.260 | -0.208 | 0.729 | 2.76E-01 | 0.503 | -0.450 | 1.456 | 3.05E-01 | 0.086 | -0.244 | 0.417 | 6.08E-01 |
|  |  |  |  |  | Banks of the Superior Temporal Sulcus | 0.167 | -0.170 | 0.503 | 3.32E-01 | 0.216 | -1.512 | 1.943 | 8.07E-01 | 0.276 | -0.209 | 0.761 | 2.64E-01 | 0.693 | -0.497 | 1.882 | 2.58E-01 | 0.171 | -0.161 | 0.503 | 3.13E-01 |

| **Table S7. MVMR analyses showed genetically predicted effects of cortical SA on adult cEF** | | | | | | | | | |
| --- | --- | --- | --- | --- | --- | --- | --- | --- | --- |
| **Exposure** | | | **Outcome** | **Conditional F** | **Q_strength** | **Q_valid *P-*value** | **dIVW Estimate** | **dIVW 95% CI** | **dIVW Condition** |
| Total brain | surface area |  | cEF | 0.23 | 19.42 | 0.576 | 0.032 | [0.024, 0.041] | 120.97 |
| Frontal | surface area | Rostral Anterior Cingulate |  | 0.73 | 60.69 | 0.576 | 0.029 | [0.020, 0.037] | 104.31 |
|  |  | Caudal Anterior Cingulate |  | 0.34 | 28.34 | 0.576 | 0.034 | [0.024, 0.044] | 75.8 |
|  |  | Superior Frontal |  | 0.3 | 24.82 | 0.576 | 0.033 | [0.024, 0.043] | 92.14 |
|  |  | Pars Orbitalis |  | 0.63 | 51.98 | 0.576 | 0.032 | [0.022, 0.042] | 80.97 |
| Temporal | surface area | Superior Temporal |  | 0.65 | 53.57 | 0.576 | 0.032 | [0.023, 0.040] | 98.21 |
|  |  | Banks of the Superior Temporal Sulcus |  | 0.73 | 60.42 | 0.576 | 0.038 | [0.027, 0.048] | 62.42 |

| **Table S8. Genetic correlations between infant HC and cortical SA** | | | | | | | |
| --- | --- | --- | --- | --- | --- | --- | --- |
| **Phenotype1** | | | **Phenotype2** | ***Genetic correlation*** | **SE** | ***Z*** | ***P-*value** |
|  |  |  |  |  |  |  |  |
| Total brain | surface area |  | infant HC | 0.803 | 0.092 | 8.704 | 3.21E-18 |
| Frontal | surface area | Rostral Anterior Cingulate | infant HC | 0.759 | 0.110 | 6.885 | 5.78E-12 |
|  |  | Caudal Anterior Cingulate | infant HC | 0.665 | 0.124 | 5.373 | 7.76E-08 |
|  |  | Superior Frontal | infant HC | 0.794 | 0.094 | 8.404 | 4.32E-17 |
|  |  | Pars Orbitalis | infant HC | 0.599 | 0.115 | 5.222 | 1.77E-07 |
| Temporal | surface area | Superior Temporal | infant HC | 0.648 | 0.100 | 6.450 | 1.12E-10 |
|  |  | Banks of the Superior Temporal Sulcus | infant HC | 0.599 | 0.101 | 5.958 | 2.55E-09 |

| **Table S9. Bidirectional MR estimates between infant HC and cortical SA** | | | | | | | | | | | | | | | | | | | | | | | | | |
| --- | --- | --- | --- | --- | --- | --- | --- | --- | --- | --- | --- | --- | --- | --- | --- | --- | --- | --- | --- | --- | --- | --- | --- | --- | --- |
| Note: Causal effects estimated using 5 two-sample Mendelian randomization methods. All statistical tests were two-sided. *P*-value < 3.57e-3 after Bonferroni correction was considered significant. | | | | | | | | | | | | | | | | | | | | | | | | | |
| **Exposure** | | | **Outcome** | | | **Inverse-variance weighted** | | | | **MR-Egger** | | | | **Weighted median** | | | | **Weighted mode** | | | | **MR RAPS** | | | |
|  |  |  |  |  |  | **Estimate (β)** | **95% lower CI** | **95% upper CI** | ***P-*value** | **Estimate (β)** | **95% lower CI** | **95% upper CI** | ***P-*value** | **Estimate (β)** | **95% lower CI** | **95% upper CI** | ***P-*value** | **Estimate (β)** | **95% lower CI** | **95% upper CI** | ***P-*value** | **Estimate (β)** | **95% lower CI** | **95% upper CI** | ***P-*value** |
| infant HC | | | Total brain | surface area |  | 0.238 | 0.157 | 0.319 | 8.44E-09 | 0.202 | -0.041 | 0.446 | 1.20E-01 | 0.140 | 0.030 | 0.250 | 1.25E-02 | 0.124 | -0.024 | 0.271 | 1.18E-01 | 0.250 | 0.174 | 0.327 | 1.56E-10 |
|  |  |  | Frontal | surface area | Rostral Anterior Cingulate | 0.252 | 0.180 | 0.324 | 8.65E-12 | 0.198 | -0.061 | 0.458 | 1.53E-01 | 0.216 | 0.095 | 0.337 | 4.59E-04 | 0.186 | -0.035 | 0.407 | 1.17E-01 | 0.259 | 0.170 | 0.349 | 1.24E-08 |
|  |  |  |  |  | Caudal Anterior Cingulate | 0.188 | 0.102 | 0.274 | 1.85E-05 | 0.255 | -0.011 | 0.520 | 7.62E-02 | 0.105 | -0.011 | 0.220 | 7.52E-02 | 0.086 | -0.126 | 0.299 | 4.35E-01 | 0.195 | 0.107 | 0.283 | 1.32E-05 |
|  |  |  |  |  | Superior Frontal | 0.248 | 0.184 | 0.312 | 3.31E-14 | 0.188 | -0.033 | 0.408 | 1.15E-01 | 0.226 | 0.118 | 0.333 | 3.93E-05 | 0.138 | -0.057 | 0.332 | 1.82E-01 | 0.255 | 0.171 | 0.339 | 2.42E-09 |
|  |  |  |  |  | Pars Orbitalis | 0.165 | 0.066 | 0.265 | 1.13E-03 | 0.087 | -0.195 | 0.370 | 5.53E-01 | 0.154 | 0.033 | 0.275 | 1.26E-02 | 0.156 | -0.077 | 0.390 | 2.07E-01 | 0.175 | 0.089 | 0.261 | 6.92E-05 |
|  |  |  | Temporal | surface area | Superior Temporal | 0.259 | 0.185 | 0.334 | 1.03E-11 | 0.280 | 0.050 | 0.511 | 2.85E-02 | 0.155 | 0.038 | 0.273 | 9.67E-03 | 0.123 | -0.071 | 0.318 | 2.29E-01 | 0.268 | 0.182 | 0.354 | 9.74E-10 |
|  |  |  |  |  | Banks of the Superior Temporal Sulcus | 0.230 | 0.145 | 0.316 | 1.13E-07 | 0.183 | -0.087 | 0.454 | 1.97E-01 | 0.164 | 0.055 | 0.273 | 3.19E-03 | 0.112 | -0.098 | 0.323 | 3.08E-01 | 0.242 | 0.160 | 0.324 | 8.59E-09 |
| Total brain | surface area |  | infant HC | | | 0.288 | 0.198 | 0.378 | 3.38E-10 | 0.240 | -0.044 | 0.524 | 1.09E-01 | 0.180 | 0.040 | 0.320 | 1.19E-02 | 0.113 | -0.178 | 0.404 | 4.52E-01 | 0.298 | 0.198 | 0.398 | 6.00E-09 |
| Frontal | surface area | Rostral Anterior Cingulate |  |  |  | 0.234 | 0.138 | 0.330 | 1.86E-06 | 0.114 | -0.199 | 0.427 | 4.82E-01 | 0.154 | 0.026 | 0.282 | 1.87E-02 | 0.117 | -0.136 | 0.370 | 3.75E-01 | 0.243 | 0.144 | 0.343 | 1.59E-06 |
|  |  | Caudal Anterior Cingulate |  |  |  | 0.138 | 0.018 | 0.259 | 2.37E-02 | -0.122 | -0.436 | 0.192 | 4.58E-01 | 0.099 | -0.054 | 0.253 | 2.04E-01 | 0.075 | -0.157 | 0.307 | 5.35E-01 | 0.145 | 0.030 | 0.261 | 1.37E-02 |
|  |  | Superior Frontal |  |  |  | 0.143 | 0.048 | 0.237 | 3.08E-03 | 0.002 | -0.283 | 0.288 | 9.88E-01 | 0.110 | -0.042 | 0.262 | 1.55E-01 | 0.095 | -0.143 | 0.332 | 4.44E-01 | 0.147 | 0.029 | 0.265 | 1.49E-02 |
|  |  | Pars Orbitalis |  |  |  | 0.127 | 0.025 | 0.229 | 1.50E-02 | -0.028 | -0.325 | 0.268 | 8.54E-01 | 0.113 | -0.025 | 0.251 | 1.08E-01 | 0.053 | -0.181 | 0.287 | 6.61E-01 | 0.132 | 0.025 | 0.239 | 1.54E-02 |
| Temporal | surface area | Superior Temporal |  |  |  | 0.314 | 0.237 | 0.391 | 9.99E-16 | 0.624 | 0.282 | 0.967 | 1.36E-03 | 0.244 | 0.121 | 0.366 | 9.50E-05 | 0.159 | -0.106 | 0.425 | 2.49E-01 | 0.324 | 0.235 | 0.413 | 9.18E-13 |
|  |  | Banks of the Superior Temporal Sulcus |  |  |  | 0.118 | -0.009 | 0.246 | 6.85E-02 | -0.154 | -0.534 | 0.227 | 4.40E-01 | 0.113 | -0.049 | 0.274 | 1.71E-01 | -0.115 | -0.420 | 0.190 | 4.69E-01 | 0.126 | 0.015 | 0.237 | 2.63E-02 |

| **Table S10. MVMR analyses showed genetically predicted effects of cortical SA on infant HC** | | | | | | | | | |
| --- | --- | --- | --- | --- | --- | --- | --- | --- | --- |
| **Exposure** | | | **Outcome** | **Conditional F** | **Q_strength** | **Q_valid *P-*value** | **dIVW Estimate** | **dIVW 95% CI** | **dIVW Condition** |
| Total brain | surface area |  | infant HC | 0.36 | 23.08 | 0.815 | 0.327 | [0.246, 0.408] | 133.2 |
| Frontal | surface area | Rostral Anterior Cingulate |  | 1.34 | 87.17 | 0.815 | 0.31 | [0.227, 0.392] | 101.86 |
|  |  | Superior Frontal |  | 0.51 | 33.4 | 0.815 | 0.308 | [0.226, 0.391] | 112.96 |
| Temporal | surface area | Superior Temporal |  | 0.81 | 52.55 | 0.815 | 0.316 | [0.239, 0.393] | 118.28 |

| **Table S11. Mediation analyses of the pathway from infant HC to adult cEF via cortical SA** | | | | | | | | |
| --- | --- | --- | --- | --- | --- | --- | --- | --- |
| **exposure** | **mediator** | **outcome** | **Total effect（95%CI）** | **Direct effect A（95%CI）** | **Direct effect B（95%CI）** | **Mediation effect（95%CI）** | ***P-*value** | **mediation proportion**  **(95% CI)** |
| infant HC | Total brain | cEF | 0.026 [0.016, 0.035] | 0.238[0.157, 0.319] | 0.028 [0.016, 0.040] | 0.007 [0.003, 0.010] | 3.00E-04 | 0.260[0.123, 0.506] |
| infant HC | Rostral Anterior Cingulate | cEF | 0.026 [0.016, 0.035] | 0.252[0.180, 0.324] | 0.018 [0.010, 0.027] | 0.005 [0.002, 0.007] | 5.00E-04 | 0.180 [0.080, 0.352] |
| infant HC | Caudal Anterior Cingulate | cEF | 0.026 [0.016, 0.035] | 0.188[0.102, 0.274] | 0.031[0.016, 0.046] | 0.006[0.002, 0.010] | 3.20E-03 | 0.227[0.088, 0.475] |
| infant HC | Superior Frontal | cEF | 0.026 [0.016, 0.035] | 0.248[0.184, 0.312] | 0.026 [0.0140, 0.039] | 0.007 [0.003, 0.010] | 3.00E-04 | 0.256[0.118, 0.495] |
| infant HC | Pars Orbitalis | cEF | 0.026 [0.016, 0.035] | 0.165[0.066, 0.265] | 0.029[0.016, 0.042] | 0.005[0.001, 0.008] | 8.90E-03 | 0.188[0.060, 0.411] |
| infant HC | Superior Temporal | cEF | 0.026 [0.016, 0.035] | 0.259 [0.185, 0.334] | 0.019[0.008, 0.030] | 0.005[0.002, 0.008] | 3.30E-03 | 0.193[0.070, 0.396] |
| infant HC | Banks of the Superior Temporal Sulcus | cEF | 0.026 [0.016, 0.035] | 0.230[0.145, 0.316] | 0.033 [0.020, 0.046] | 0.008[0.003, 0.012] | 3.00E-04 | 0.294[0.138, 0.575] |

| **Table S12. IVs used for each exposure-outcome pairs in the MR analyses.** | | | | | | | |
| --- | --- | --- | --- | --- | --- | --- | --- |
| **Exposure** | | | **Outcome** | | | **No. of instrumental variables** | **Instrumental variables** |
| infant HC | | | cEF | | | 20 | rs1014098,rs10846487,rs10883848,rs12513756,rs143094271,rs150266910,rs2106016,rs2448415,rs261752,rs3134614,rs4674101,rs503783,rs6016511,rs62580922,rs72952297,rs72979897,rs7658192,rs7792211,rs8756,rs9853018 |
| cEF | | | infant HC | | | 61 | rs10010325,rs10495508,rs1079596,rs10797119,rs1080026,rs10964203,rs11121053,rs11202929,rs112096788,rs11252451,rs11583896,rs11660554,rs11707940,rs11733006,rs11959119,rs12172958,rs12707117,rs12894136,rs1388744,rs139507,rs1567154,rs17460320,rs17549128,rs1956001,rs2001607,rs2238057,rs2253855,rs2271763,rs2280141,rs235924,rs2470167,rs2693672,rs27989,rs2901437,rs309171,rs34885420,rs35214871,rs36120363,rs4500960,rs4588192,rs4727815,rs528483,rs55658584,rs58142369,rs59048764,rs61738821,rs62146417,rs6559660,rs6699558,rs6938696,rs709692,rs72973055,rs7687673,rs7716567,rs7725131,rs79984539,rs8023459,rs812603,rs9748337,rs9827246,rs9832922 |
| Total brain | surface area |  | cEF | | | 25 | rs10049894,rs10251751,rs10512789,rs10747968,rs10878349,rs12333007,rs12452834,rs12630663,rs16894917,rs17364659,rs17736216,rs2195243,rs284526,rs325700,rs3737880,rs41277471,rs67297650,rs71541906,rs72959469,rs7570830,rs77609904,rs7975351,rs9369294,rs9428576,rs9933611 |
| Frontal | surface area | Medial Orbitofrontal |  |  |  | 24 | rs10485212,rs11249701,rs11630761,rs1257169,rs12624716,rs12932297,rs16828204,rs1695511,rs2330917,rs2423238,rs2804939,rs325700,rs41277471,rs4665109,rs61745903,rs6794453,rs71541906,rs72884643,rs7312464,rs73735038,rs74840505,rs77652558,rs7778997,rs9810789 |
|  |  | Rostral Anterior Cingulate |  |  |  | 25 | rs116218963,rs12452834,rs1274522,rs12954474,rs177080,rs1992237,rs2540926,rs2932537,rs2971655,rs35008190,rs41277471,rs6663947,rs6850715,rs6994837,rs7195695,rs7531629,rs76501145,rs7778997,rs8017620,rs80290479,rs9369294,rs9427061,rs9568829,rs9616209,rs9776085 |
|  |  | Caudal Anterior Cingulate |  |  |  | 15 | rs10878349,rs12096763,rs12129970,rs12452834,rs12926634,rs143729942,rs145394641,rs178889,rs2271386,rs2509765,rs267135,rs58574713,rs75141912,rs929387,rs9568829 |
|  |  | Superior Frontal |  |  |  | 15 | rs10483213,rs10788183,rs10878349,rs116034822,rs12630663,rs17865692,rs2195243,rs284526,rs331959,rs34130495,rs60618359,rs6606611,rs77037886,rs80290479,rs9369294 |
|  |  | Pars Orbitalis |  |  |  | 17 | rs10446454,rs10802064,rs10932876,rs11114027,rs142465506,rs2090994,rs2177470,rs2271386,rs2839306,rs35969387,rs4869897,rs7250421,rs7312464,rs73145937,rs74700822,rs7823271,rs905955 |
| Parietal | surface area | Precuneus |  |  |  | 22 | rs10187724,rs10188396,rs10792025,rs11111293,rs114190573,rs1163075,rs1325995,rs149255261,rs2540926,rs28517651,rs34900806,rs41345247,rs4455881,rs61996723,rs660633,rs7209131,rs7601767,rs76472207,rs78419480,rs7902527,rs858993,rs968427 |
|  |  | Superior Parietal |  |  |  | 27 | rs10032097,rs10109434,rs10477683,rs111940480,rs112193068,rs1163075,rs116623451,rs11671326,rs11701091,rs1512818,rs1978302,rs36035924,rs41402,rs4455881,rs4838960,rs4924345,rs6065290,rs62046598,rs624290,rs6720027,rs67328311,rs7515720,rs7635829,rs877235,rs9637525,rs9788147,rs999724 |
|  |  | Posterior Cingulate |  |  |  | 17 | rs10744766,rs10788109,rs13249751,rs1847042,rs2293446,rs28397238,rs2881400,rs58691096,rs6899663,rs6952255,rs73176834,rs73230793,rs73403241,rs7601767,rs76501145,rs78303398,rs7998521 |
| Temporal | surface area | Entorhinal |  |  |  | 14 | rs11746026,rs1807198,rs190554442,rs247786,rs2913866,rs59846446,rs62173537,rs6791514,rs7121813,rs7172916,rs72800968,rs746729,rs75236952,rs78865504 |
|  |  | Superior Temporal |  |  |  | 22 | rs10878349,rs113542967,rs1247485,rs1257169,rs12630663,rs12694390,rs1527967,rs1561369,rs178481,rs34464850,rs34719936,rs41277471,rs4314272,rs4625112,rs6555938,rs67297650,rs6961064,rs76650567,rs7676700,rs77682716,rs8181632,rs9372832 |
|  |  | Banks of the Superior Temporal Sulcus |  |  |  | 15 | rs10811554,rs11188356,rs113601403,rs11710049,rs12631240,rs17776733,rs2147063,rs2237430,rs2303242,rs2832501,rs35597264,rs682780,rs7603966,rs829829,rs9669278 |
| cEF | | | Total brain | surface area |  | 62 | rs10010325,rs10495508,rs1079596,rs10797119,rs1080026,rs10964203,rs11121053,rs11130017,rs11202929,rs112096788,rs11583896,rs11660554,rs11707940,rs11733006,rs11959119,rs12172958,rs12707117,rs12894136,rs1388744,rs139507,rs1567154,rs17460320,rs17549128,rs1956001,rs2001607,rs2238057,rs2253855,rs2271763,rs2280141,rs235924,rs2470167,rs27989,rs2901437,rs309171,rs34885420,rs35214871,rs36120363,rs4500960,rs4588192,rs4727815,rs528483,rs55658584,rs58142369,rs59048764,rs6095319,rs61738821,rs6559660,rs6699558,rs6938696,rs709692,rs72973055,rs7687673,rs7716567,rs7725131,rs7994762,rs79984539,rs8023459,rs805360,rs812603,rs9748337,rs9827246,rs9832922 |
|  |  |  | Frontal | surface area | Medial Orbitofrontal | 62 | rs10010325,rs10495508,rs1079596,rs10797119,rs1080026,rs10964203,rs11121053,rs11130017,rs11202929,rs112096788,rs11252451,rs11583896,rs11660554,rs11733006,rs11959119,rs12172958,rs12707117,rs12894136,rs1388744,rs139507,rs1567154,rs17460320,rs17549128,rs1956001,rs2001607,rs2238057,rs2253855,rs2271763,rs2280141,rs235924,rs2470167,rs2693672,rs27989,rs309171,rs34885420,rs35214871,rs36120363,rs4500960,rs4588192,rs4727815,rs528483,rs55658584,rs58142369,rs59048764,rs6095319,rs61738821,rs6559660,rs6699558,rs6938696,rs709692,rs72973055,rs7687673,rs7716567,rs7725131,rs7994762,rs79984539,rs8023459,rs805360,rs812603,rs9748337,rs9827246,rs9832922 |
|  |  |  |  |  | Rostral Anterior Cingulate | 62 | rs10010325,rs10495508,rs1079596,rs10797119,rs1080026,rs10964203,rs11121053,rs11130017,rs11202929,rs112096788,rs11252451,rs11583896,rs11660554,rs11707940,rs11733006,rs11959119,rs12172958,rs12707117,rs12894136,rs1388744,rs139507,rs1567154,rs17460320,rs17549128,rs1956001,rs2001607,rs2238057,rs2271763,rs2280141,rs235924,rs2470167,rs27989,rs2901437,rs309171,rs34885420,rs35214871,rs36120363,rs4500960,rs4588192,rs4727815,rs528483,rs55658584,rs58142369,rs59048764,rs6095319,rs61738821,rs6559660,rs6699558,rs6938696,rs709692,rs72973055,rs7687673,rs7716567,rs7725131,rs7994762,rs79984539,rs8023459,rs805360,rs812603,rs9748337,rs9827246,rs9832922 |
|  |  |  |  |  | Caudal Anterior Cingulate | 62 | rs10010325,rs10495508,rs1079596,rs10797119,rs1080026,rs11121053,rs11130017,rs11202929,rs112096788,rs11252451,rs11583896,rs11660554,rs11707940,rs11733006,rs11959119,rs12172958,rs12707117,rs12894136,rs1388744,rs139507,rs1567154,rs17460320,rs17549128,rs1956001,rs2001607,rs2238057,rs2253855,rs2271763,rs2280141,rs235924,rs2470167,rs2693672,rs27989,rs2901437,rs309171,rs34885420,rs35214871,rs36120363,rs4588192,rs4727815,rs528483,rs55658584,rs58142369,rs59048764,rs6095319,rs61738821,rs6559660,rs6699558,rs6938696,rs709692,rs72973055,rs7687673,rs7716567,rs7725131,rs7994762,rs79984539,rs8023459,rs805360,rs812603,rs9748337,rs9827246,rs9832922 |
|  |  |  |  |  | Superior Frontal | 63 | rs10010325,rs10495508,rs1079596,rs10797119,rs1080026,rs10964203,rs11121053,rs11130017,rs11202929,rs112096788,rs11252451,rs11583896,rs11660554,rs11707940,rs11733006,rs11959119,rs12172958,rs12707117,rs12894136,rs1388744,rs139507,rs1567154,rs17460320,rs17549128,rs1956001,rs2001607,rs2238057,rs2253855,rs2271763,rs2280141,rs235924,rs2470167,rs27989,rs2901437,rs309171,rs34885420,rs35214871,rs36120363,rs4500960,rs4588192,rs4727815,rs528483,rs55658584,rs58142369,rs59048764,rs6095319,rs61738821,rs6559660,rs6699558,rs6938696,rs709692,rs72973055,rs7687673,rs7716567,rs7725131,rs7994762,rs79984539,rs8023459,rs805360,rs812603,rs9748337,rs9827246,rs9832922 |
|  |  |  |  |  | Pars Orbitalis | 63 | rs10010325,rs10495508,rs1079596,rs10797119,rs1080026,rs10964203,rs11121053,rs11130017,rs11202929,rs112096788,rs11252451,rs11583896,rs11660554,rs11707940,rs11733006,rs11959119,rs12172958,rs12707117,rs12894136,rs1388744,rs139507,rs1567154,rs17460320,rs17549128,rs1956001,rs2001607,rs2238057,rs2253855,rs2271763,rs2280141,rs235924,rs2470167,rs27989,rs2901437,rs309171,rs34885420,rs35214871,rs36120363,rs4500960,rs4588192,rs4727815,rs528483,rs55658584,rs58142369,rs59048764,rs6095319,rs61738821,rs6559660,rs6699558,rs6938696,rs709692,rs72973055,rs7687673,rs7716567,rs7725131,rs7994762,rs79984539,rs8023459,rs805360,rs812603,rs9748337,rs9827246,rs9832922 |
|  |  |  | Parietal | surface area | Precuneus | 59 | rs10010325,rs10495508,rs1079596,rs10797119,rs1080026,rs11121053,rs11130017,rs112096788,rs11583896,rs11660554,rs11707940,rs11733006,rs11959119,rs12172958,rs12707117,rs12894136,rs1388744,rs139507,rs1567154,rs17460320,rs17549128,rs1956001,rs2001607,rs2238057,rs2253855,rs2271763,rs2280141,rs235924,rs2470167,rs27989,rs2901437,rs309171,rs34885420,rs35214871,rs36120363,rs4500960,rs4588192,rs4727815,rs528483,rs55658584,rs58142369,rs59048764,rs6095319,rs61738821,rs6559660,rs6699558,rs6938696,rs709692,rs72973055,rs7687673,rs7716567,rs7725131,rs7994762,rs8023459,rs805360,rs812603,rs9748337,rs9827246,rs9832922 |
|  |  |  |  |  | Superior Parietal | 62 | rs10010325,rs10495508,rs1079596,rs10797119,rs1080026,rs10964203,rs11121053,rs11130017,rs11202929,rs112096788,rs11583896,rs11660554,rs11707940,rs11733006,rs12172958,rs12707117,rs12894136,rs1388744,rs139507,rs1567154,rs17460320,rs17549128,rs1956001,rs2001607,rs2238057,rs2253855,rs2271763,rs2280141,rs235924,rs2470167,rs2693672,rs27989,rs2901437,rs309171,rs34885420,rs35214871,rs36120363,rs4500960,rs4588192,rs4727815,rs528483,rs55658584,rs58142369,rs59048764,rs6095319,rs61738821,rs6559660,rs6699558,rs6938696,rs709692,rs72973055,rs7687673,rs7716567,rs7725131,rs7994762,rs79984539,rs8023459,rs805360,rs812603,rs9748337,rs9827246,rs9832922 |
|  |  |  |  |  | Posterior Cingulate | 61 | rs10010325,rs10495508,rs1079596,rs10797119,rs1080026,rs10964203,rs11121053,rs11130017,rs11202929,rs112096788,rs11252451,rs11583896,rs11660554,rs11707940,rs11733006,rs11959119,rs12172958,rs12707117,rs12894136,rs1388744,rs139507,rs1567154,rs17460320,rs17549128,rs1956001,rs2001607,rs2238057,rs2253855,rs2271763,rs235924,rs2470167,rs27989,rs2901437,rs309171,rs34885420,rs35214871,rs36120363,rs4500960,rs4588192,rs4727815,rs528483,rs55658584,rs58142369,rs59048764,rs6095319,rs61738821,rs6559660,rs6699558,rs6938696,rs709692,rs72973055,rs7687673,rs7716567,rs7725131,rs7994762,rs79984539,rs8023459,rs805360,rs812603,rs9748337,rs9832922 |
|  |  |  | Temporal | surface area | Entorhinal | 63 | rs10010325,rs10495508,rs1079596,rs10797119,rs1080026,rs10964203,rs11121053,rs11130017,rs11202929,rs112096788,rs11252451,rs11583896,rs11660554,rs11707940,rs11733006,rs11959119,rs12172958,rs12707117,rs12894136,rs1388744,rs139507,rs1567154,rs17460320,rs17549128,rs1956001,rs2001607,rs2238057,rs2253855,rs2271763,rs2280141,rs235924,rs2470167,rs2693672,rs27989,rs2901437,rs309171,rs34885420,rs35214871,rs36120363,rs4500960,rs4588192,rs4727815,rs528483,rs55658584,rs58142369,rs59048764,rs6095319,rs61738821,rs6559660,rs6699558,rs6938696,rs709692,rs72973055,rs7687673,rs7716567,rs7725131,rs7994762,rs79984539,rs8023459,rs805360,rs812603,rs9748337,rs9827246 |
|  |  |  |  |  | Superior Temporal | 61 | rs10010325,rs10495508,rs1079596,rs10797119,rs1080026,rs10964203,rs11121053,rs11130017,rs11202929,rs112096788,rs11252451,rs11583896,rs11660554,rs11707940,rs11733006,rs11959119,rs12172958,rs12707117,rs12894136,rs1388744,rs139507,rs1567154,rs17460320,rs17549128,rs1956001,rs2001607,rs2238057,rs2253855,rs235924,rs2470167,rs27989,rs2901437,rs309171,rs34885420,rs35214871,rs36120363,rs4500960,rs4588192,rs4727815,rs528483,rs55658584,rs58142369,rs59048764,rs6095319,rs61738821,rs6559660,rs6699558,rs6938696,rs709692,rs72973055,rs7687673,rs7716567,rs7725131,rs7994762,rs79984539,rs8023459,rs805360,rs812603,rs9748337,rs9827246,rs9832922 |
|  |  |  |  |  | Banks of the Superior Temporal Sulcus | 63 | rs10010325,rs10495508,rs1079596,rs10797119,rs1080026,rs10964203,rs11121053,rs11130017,rs11202929,rs112096788,rs11252451,rs11583896,rs11660554,rs11707940,rs11733006,rs11959119,rs12172958,rs12707117,rs12894136,rs1388744,rs139507,rs1567154,rs17460320,rs17549128,rs1956001,rs2001607,rs2238057,rs2253855,rs2271763,rs2280141,rs235924,rs2470167,rs2693672,rs27989,rs2901437,rs309171,rs34885420,rs35214871,rs36120363,rs4500960,rs4588192,rs4727815,rs528483,rs55658584,rs58142369,rs59048764,rs6095319,rs61738821,rs6559660,rs6699558,rs6938696,rs709692,rs72973055,rs7687673,rs7716567,rs7725131,rs7994762,rs79984539,rs8023459,rs805360,rs812603,rs9748337,rs9827246 rs9832922 |
| infant HC | | | Total brain | surface area |  | 20 | rs10846487,rs12350281,rs12513756,rs143094271,rs150266910,rs17223831,rs2448415,rs261752,rs3134614,rs4674101,rs503783,rs6016511,rs62580922,rs72952297,rs72979897,rs76065384,rs7658192,rs77535478,rs7792211,rs9853018 |
|  |  |  | Frontal | surface area | Rostral Anterior Cingulate | 19 | rs10846487,rs10883848,rs12513756,rs143094271,rs150266910,rs17223831,rs2106016,rs2448415,rs3134614,rs4674101,rs503783,rs6016511,rs62580922,rs72979897,rs76065384,rs7658192,rs77535478,rs7792211,rs9853018 |
|  |  |  |  |  | Caudal Anterior Cingulate | 20 | rs1014098,rs10846487,rs12350281,rs12513756,rs143094271,rs150266910,rs17223831,rs2448415,rs261752,rs3134614,rs4674101,rs503783,rs6016511,rs62580922,rs72979897,rs76065384,rs7658192,rs77535478,rs7792211,rs9853018 |
|  |  |  |  |  | Superior Frontal | 19 | rs1014098,rs10846487,rs12350281,rs12513756,rs143094271,rs150266910,rs17223831,rs2448415,rs261752,rs3134614,rs6016511,rs62580922,rs72952297,rs72979897,rs76065384,rs7658192,rs77535478,rs7792211,rs9853018 |
|  |  |  |  |  | Pars Orbitalis | 18 | rs12350281,rs12513756,rs143094271,rs150266910,rs17223831,rs2448415,rs261752,rs4674101,rs503783,rs6016511,rs62580922,rs72952297,rs72979897,rs76065384,rs7658192,rs77535478,rs7792211,rs9853018 |
|  |  |  | Temporal | surface area | Superior Temporal | 20 | rs10846487,rs12350281,rs12513756,rs143094271,rs150266910,rs17223831,rs2448415,rs261752,rs3134614,rs4674101,rs503783,rs6016511,rs62580922,rs72952297,rs72979897,rs76065384,rs7658192,rs77535478,rs7792211,rs9853018 |
|  |  |  |  |  | Banks of the Superior Temporal Sulcus | 23 | rs1014098,rs10846487,rs10883848,rs12350281,rs12513756,rs143094271,rs150266910,rs17223831,rs2106016,rs2448415,rs261752,rs3134614,rs4674101,rs503783,rs6016511,rs62580922,rs72952297,rs72979897,rs76065384,rs7658192,rs77535478,rs7792211,rs9853018 |
| Total brain | surface area |  | infant HC | | | 29 | rs10049894,rs10251751,rs10512789,rs10747968,rs116623451,rs11705230,rs12452834,rs12630663,rs16894917,rs17364659,rs17736216,rs2195243,rs2301718,rs284526,rs284849,rs325700,rs3737880,rs41277471,rs4730073,rs6555948,rs67297650,rs72959469,rs7477335,rs78158162,rs7975351,rs8137902,rs9369294,rs9428576,rs9933611 |
| Frontal | surface area | Rostral Anterior Cingulate |  |  |  | 23 | rs116218963,rs12452834,rs1274522,rs12954474,rs144513199,rs177080,rs1921610,rs1992237,rs2932537,rs2971655,rs41277471,rs6663947,rs6850715,rs6994837,rs7195695,rs76501145,rs7778997,rs8017620,rs80290479,rs9369294,rs9427061,rs9568829,rs9616209 |
|  |  | Caudal Anterior Cingulate |  |  |  | 17 | rs11176822,rs12096763,rs12129970,rs12452834,rs12926634,rs143729942,rs145394641,rs178889,rs2509765,rs2871962,rs58574713,rs6675870,rs72638516,rs75141912,rs7601767,rs929387,rs9568829 |
|  |  | Superior Frontal |  |  |  | 19 | rs10483213,rs116034822,rs116623451,rs12599271,rs12630663,rs144513199,rs17865692,rs2188185,rs2195243,rs284526,rs331959,rs34130495,rs60618359,rs6606611,rs72884643,rs77037886,rs80290479,rs9369294,rs9909861 |
|  |  | Pars Orbitalis |  |  |  | 20 | rs10446454,rs10802064,rs10932876,rs11114027,rs113931321,rs1243189,rs141734354,rs142465506,rs2090994,rs2177470,rs2839306,rs35969387,rs4869897,rs6865689,rs7250421,rs72690863,rs73145937,rs74700822,rs7823271,rs905955 |
| Temporal | surface area | Superior Temporal |  |  |  | 29 | rs113542967,rs11626739,rs1163249,rs11887499,rs1197661,rs1247485,rs1257169,rs12630663,rs12694390,rs1527967,rs1561369,rs178481,rs2969,rs34059176,rs34464850,rs34719936,rs41277471,rs4314272,rs4625112,rs4747437,rs6555938,rs67297650,rs6961064,rs7514834,rs76650567,rs7676700,rs77682716,rs78158162,rs8181632 |
|  |  | Banks of the Superior Temporal Sulcus |  |  |  | 18 | rs10811554,rs10995252,rs11188356,rs113601403,rs11710049,rs12101308,rs12458048,rs12631240,rs2147063,rs2237430,rs2303242,rs2832501,rs28722191,rs30101,rs3935357,rs682780,rs7603966,rs829829 |
| **MVMR** | | | cEF | | | 83 | rs10049894,rs10446454,rs10512789,rs10747968,rs10788183,rs10802064,rs10878349,rs10932876,rs11114027,rs11188356,rs113601403,rs116034822,rs116218963,rs11710049,rs12333007,rs12452834,rs1247485,rs1257169,rs12631240,rs1274522,rs12954474,rs142465506,rs143729942,rs1561369,rs177080,rs17736216,rs17776733,rs178481,rs17865692,rs178889,rs1992237,rs2195243,rs2237430,rs2509765,rs2540926,rs267135,rs2832501,rs2839306,rs2932537,rs2971655,rs325700,rs34130495,rs34464850,rs34719936,rs41277471,rs4314272,rs4625112,rs4869897,rs58574713,rs6555938,rs6606611,rs67297650,rs682780,rs6850715,rs7195695,rs7250421,rs72959469,rs73145937,rs74700822,rs75141912,rs7531629,rs7570830,rs7603966,rs76501145,rs76650567,rs7676700,rs77037886,rs77609904,rs7778997,rs7823271,rs8017620,rs80290479,rs8181632,rs829829,rs905955,rs9369294,rs9372832,rs9427061,rs9428576,rs9568829,rs9616209,rs9776085,rs9933611 |
| Total brain | surface area |  |  |  |  |  |  |
| Frontal | surface area | Rostral Anterior Cingulate |  |  |  |  |  |
|  |  | Caudal Anterior Cingulate |  |  |  |  |  |
|  |  | Superior Frontal |  |  |  |  |  |
|  |  | Pars Orbitalis |  |  |  |  |  |
| Temporal | surface area | Superior Temporal |  |  |  |  |  |
|  |  | Banks of the Superior Temporal Sulcus |  |  |  |  |  |
| Total brain | surface area |  | infant HC | | | 65 | rs10049894,rs10512789,rs10747968,rs116034822,rs116218963,rs11626739,rs1163249,rs116623451,rs11705230,rs11887499,rs1197661,rs1247485,rs1257169,rs12630663,rs1274522,rs12954474,rs144513199,rs1527967,rs1561369,rs177080,rs17736216,rs178481,rs17865692,rs1921610,rs1992237,rs2188185,rs2195243,rs2301718,rs284526,rs2932537,rs2969,rs2971655,rs325700,rs331959,rs34059176,rs34130495,rs34464850,rs34719936,rs41277471,rs4314272,rs4625112,rs4730073,rs6555938,rs6606611,rs67297650,rs6994837,rs7195695,rs72884643,rs72959469,rs7477335,rs76501145,rs76650567,rs7676700,rs77037886,rs77682716,rs7778997,rs8017620,rs80290479,rs8137902,rs8181632,rs9369294,rs9427061,rs9428576 rs9568829,rs9933611 |
| Frontal | surface area | Rostral Anterior Cingulate |  |  |  |  |  |
|  |  | Superior Frontal |  |  |  |  |  |
| Temporal | surface area | Superior Temporal |  |  |  |  |  |

| **Table S13. Sensitivity analysis results for all exposure-outcome pairs in MR analyses.** | | | | | | | | |
| --- | --- | --- | --- | --- | --- | --- | --- | --- |
| **Exposure** | | | **Outcome** | | | **MR-PRESSO global test (*P-*value)** | **Cochran’s Q (*P-*value)** | **MR-Egger intercept (*P-*value)** |
| infant HC | | | cEF | | | 3.82E-01 | 3.35E-01 | 6.20E-01 |
| cEF | | | infant HC | | | 2.41E-01 | 2.41E-01 | 5.88E-01 |
| Total brain | surface area |  | cEF | | | 2.69E-01 | 2.31E-01 | 5.60E-01 |
| Frontal | surface area | Medial Orbitofrontal |  |  |  | 1.95E-01 | 1.92E-01 | 7.99E-01 |
|  |  | Rostral Anterior Cingulate |  |  |  | 7.12E-01 | 6.99E-01 | 7.16E-01 |
|  |  | Caudal Anterior Cingulate |  |  |  | 1.08E-01 | 5.94E-02 | 4.38E-02 |
|  |  | Superior Frontal |  |  |  | 6.55E-01 | 6.31E-01 | 8.03E-01 |
|  |  | Pars Orbitalis |  |  |  | 3.04E-01 | 2.58E-01 | 9.96E-01 |
| Parietal | surface area | Precuneus |  |  |  | 1.62E-01 | 1.71E-01 | 2.81E-01 |
|  |  | Superior Parietal |  |  |  | 2.21E-01 | 1.95E-01 | 8.09E-02 |
|  |  | Posterior Cingulate |  |  |  | 4.65E-01 | 5.13E-01 | 7.32E-01 |
| Temporal | surface area | Entorhinal |  |  |  | 4.30E-01 | 4.22E-01 | 1.89E-01 |
|  |  | Superior Temporal |  |  |  | 1.78E-01 | 1.52E-01 | 5.45E-01 |
|  |  | Banks of the Superior Temporal Sulcus |  |  |  | 4.15E-01 | 3.44E-01 | 2.25E-01 |
| cEF | | | Total brain | surface area |  | 3.79E-01 | 3.80E-01 | 9.06E-01 |
|  |  |  | Frontal | surface area | Medial Orbitofrontal | 2.40E-01 | 2.25E-01 | 8.26E-01 |
|  |  |  |  |  | Rostral Anterior Cingulate | 1.85E-01 | 1.87E-01 | 8.66E-01 |
|  |  |  |  |  | Caudal Anterior Cingulate | 2.33E-01 | 2.49E-01 | 3.48E-01 |
|  |  |  |  |  | Superior Frontal | 3.89E-01 | 3.71E-01 | 2.43E-01 |
|  |  |  |  |  | Pars Orbitalis | 7.20E-02 | 7.47E-02 | 3.38E-01 |
|  |  |  | Parietal | surface area | Precuneus | 2.09E-01 | 2.20E-01 | 1.90E-01 |
|  |  |  |  |  | Superior Parietal | 2.17E-01 | 2.24E-01 | 9.74E-01 |
|  |  |  |  |  | Posterior Cingulate | 6.00E-02 | 5.16E-02 | 5.98E-01 |
|  |  |  | Temporal | surface area | Entorhinal | 1.06E-01 | 9.82E-02 | 4.36E-01 |
|  |  |  |  |  | Superior Temporal | 6.68E-01 | 6.74E-01 | 9.89E-01 |
|  |  |  |  |  | Banks of the Superior Temporal Sulcus | 3.52E-01 | 3.08E-01 | 9.55E-01 |
| infant HC | | | Total brain | surface area |  | 2.50E-01 | 1.55E-01 | 7.66E-01 |
|  |  |  | Frontal | surface area | Rostral Anterior Cingulate | 8.00E-01 | 6.77E-01 | 6.76E-01 |
|  |  |  |  |  | Caudal Anterior Cingulate | 4.07E-01 | 3.59E-01 | 6.08E-01 |
|  |  |  |  |  | Superior Frontal | 8.52E-01 | 8.09E-01 | 5.76E-01 |
|  |  |  |  |  | Pars Orbitalis | 1.00E-01 | 8.82E-02 | 5.72E-01 |
|  |  |  | Temporal | surface area | Superior Temporal | 6.77E-01 | 5.81E-01 | 8.51E-01 |
|  |  |  |  |  | Banks of the Superior Temporal Sulcus | 2.69E-01 | 2.03E-01 | 7.23E-01 |
| Total brain | surface area |  | infant HC | | | 6.44E-01 | 5.88E-01 | 7.31E-01 |
| Frontal | surface area | Rostral Anterior Cingulate |  |  |  | 4.16E-01 | 3.78E-01 | 4.40E-01 |
|  |  | Caudal Anterior Cingulate |  |  |  | 2.99E-01 | 2.70E-01 | 1.02E-01 |
|  |  | Superior Frontal |  |  |  | 8.06E-01 | 7.96E-01 | 3.10E-01 |
|  |  | Pars Orbitalis |  |  |  | 4.66E-01 | 4.45E-01 | 2.90E-01 |
| Temporal | surface area | Superior Temporal |  |  |  | 7.59E-01 | 6.53E-01 | 7.86E-02 |
|  |  | Banks of the Superior Temporal Sulcus |  |  |  | 1.12E-01 | 1.08E-01 | 1.58E-01 |

| **Table S14. Sensitivity analysis of primary MR estimates using a more stringent instrument selection threshold (*P*<1e-6)** | | | | | | | | | | | | | | | | | | | | | | | | | |
| --- | --- | --- | --- | --- | --- | --- | --- | --- | --- | --- | --- | --- | --- | --- | --- | --- | --- | --- | --- | --- | --- | --- | --- | --- | --- |
| Note: Causal effects estimated using 5 two-sample Mendelian randomization methods (inverse-variance weighted, MR-Egger, weighted median, weighted mode, and RAPS). All statistical tests were two-sided. | | | | | | | | | | | | | | | | | | | | | | | | | |
| **Exposure** | | | **Outcome** | | | **Inverse-variance weighted** | | | | **MR-Egger** | | | | **Weighted median** | | | | **Weighted mode** | | | | **MR RAPS** | | | |
|  |  |  |  |  |  | **Estimate (β)** | **95% lower CI** | **95% upper CI** | ***P-*value** | **Estimate (β)** | **95% lower CI** | **95% upper CI** | ***P-*value** | **Estimate (β)** | **95% lower CI** | **95% upper CI** | ***P-*value** | **Estimate (β)** | **95% lower CI** | **95% upper CI** | ***P-*value** | **Estimate (β)** | **95% lower CI** | **95% upper CI** | ***P-*value** |
| infant HC | | | cEF | | | 0.031 | 0.019 | 0.042 | 3.13E-07 | 0.019 | -0.032 | 0.070 | 4.95E-01 | 0.026 | 0.008 | 0.045 | 5.09E-03 | 0.015 | -0.015 | 0.045 | 3.59E-01 | 0.031 | 0.017 | 0.046 | 2.04E-05 |
| Total brain | surface area |  | cEF | | | 0.030 | 0.012 | 0.047 | 9.90E-04 | 0.020 | -0.056 | 0.096 | 6.16E-01 | 0.029 | 0.006 | 0.052 | 1.19E-02 | 0.031 | -0.006 | 0.067 | 1.36E-01 | 0.031 | 0.014 | 0.047 | 2.57E-04 |
| Frontal | surface area | Medial Orbitofrontal |  |  |  | 0.024 | -0.003 | 0.052 | 8.50E-02 | -0.077 | -0.169 | 0.016 | 2.45E-01 | 0.027 | 0.000 | 0.054 | 5.11E-02 | 0.036 | -0.004 | 0.075 | 1.76E-01 | 0.025 | 0.003 | 0.047 | 2.59E-02 |
|  |  | Rostral Anterior Cingulate |  |  |  | 0.019 | 0.002 | 0.036 | 2.76E-02 | 0.017 | -0.039 | 0.073 | 5.81E-01 | 0.018 | -0.005 | 0.041 | 1.33E-01 | 0.033 | -0.003 | 0.070 | 1.23E-01 | 0.020 | 0.002 | 0.038 | 2.98E-02 |
|  |  | Caudal Anterior Cingulate |  |  |  | 0.043 | 0.019 | 0.067 | 5.10E-04 | -0.058 | -0.153 | 0.036 | 3.12E-01 | 0.055 | 0.027 | 0.083 | 1.02E-04 | 0.061 | 0.016 | 0.106 | 5.67E-02 | 0.044 | 0.023 | 0.066 | 5.10E-05 |
|  |  | Superior Frontal |  |  |  | 0.025 | -0.004 | 0.055 | 9.37E-02 | -0.132 | -0.292 | 0.029 | 2.48E-01 | 0.018 | -0.012 | 0.048 | 2.45E-01 | 0.008 | -0.038 | 0.054 | 7.60E-01 | 0.026 | 0.001 | 0.052 | 4.50E-02 |
|  |  | Pars Orbitalis |  |  |  | 0.034 | 0.014 | 0.054 | 7.65E-04 | 0.017 | -0.061 | 0.095 | 6.90E-01 | 0.031 | 0.007 | 0.054 | 1.19E-02 | 0.012 | -0.031 | 0.054 | 6.11E-01 | 0.036 | 0.018 | 0.054 | 1.04E-04 |
| Parietal | surface area | Precuneus |  |  |  | -0.005 | -0.022 | 0.013 | 6.00E-01 | 0.001 | -0.089 | 0.092 | 9.81E-01 | -0.003 | -0.025 | 0.019 | 7.98E-01 | -0.027 | -0.066 | 0.012 | 2.11E-01 | -0.005 | -0.021 | 0.011 | 5.54E-01 |
|  |  | Superior Parietal |  |  |  | 0.013 | 0.001 | 0.025 | 2.97E-02 | 0.005 | -0.029 | 0.038 | 7.97E-01 | 0.011 | -0.006 | 0.027 | 2.01E-01 | 0.009 | -0.018 | 0.037 | 5.21E-01 | 0.013 | 0.001 | 0.026 | 3.74E-02 |
|  |  | Posterior Cingulate |  |  |  | -0.003 | -0.029 | 0.022 | 7.95E-01 | 0.062 | -0.004 | 0.129 | 1.61E-01 | -0.011 | -0.039 | 0.016 | 4.18E-01 | -0.017 | -0.065 | 0.030 | 5.21E-01 | -0.004 | -0.024 | 0.017 | 7.30E-01 |
| Temporal | surface area | Entorhinal |  |  |  | 0.026 | 0.003 | 0.049 | 2.78E-02 | -0.059 | -0.197 | 0.079 | 4.63E-01 | 0.031 | 0.002 | 0.059 | 3.33E-02 | 0.037 | -0.002 | 0.076 | 1.35E-01 | 0.027 | 0.005 | 0.048 | 1.37E-02 |
|  |  | Superior Temporal |  |  |  | 0.030 | 0.011 | 0.048 | 2.00E-03 | 0.001 | -0.105 | 0.107 | 9.90E-01 | 0.042 | 0.021 | 0.063 | 9.21E-05 | 0.046 | 0.012 | 0.081 | 3.40E-02 | 0.031 | 0.015 | 0.047 | 1.85E-04 |
|  |  | Banks of the Superior Temporal Sulcus |  |  |  | 0.047 | 0.031 | 0.062 | 5.24E-09 | -0.007 | -0.090 | 0.076 | 8.81E-01 | 0.047 | 0.019 | 0.076 | 1.08E-03 | 0.051 | 0.015 | 0.086 | 4.79E-02 | 0.047 | 0.023 | 0.071 | 9.85E-05 |
| infant HC | | | Total brain | surface area |  | 0.351 | 0.210 | 0.491 | 9.31E-07 | 0.187 | -0.325 | 0.699 | 5.02E-01 | 0.379 | 0.220 | 0.537 | 2.92E-06 | 0.503 | 0.213 | 0.793 | 1.15E-02 | 0.367 | 0.248 | 0.486 | 1.49E-09 |
|  |  |  | Frontal | surface area | Rostral Anterior Cingulate | 0.284 | 0.165 | 0.402 | 2.66E-06 | 0.148 | -0.287 | 0.584 | 5.30E-01 | 0.348 | 0.183 | 0.513 | 3.47E-05 | 0.406 | 0.094 | 0.719 | 3.82E-02 | 0.292 | 0.162 | 0.422 | 1.11E-05 |
|  |  |  |  |  | Caudal Anterior Cingulate | 0.282 | 0.149 | 0.415 | 3.28E-05 | 0.214 | -0.288 | 0.716 | 4.35E-01 | 0.167 | -0.013 | 0.347 | 6.96E-02 | 0.119 | -0.133 | 0.370 | 3.86E-01 | 0.292 | 0.159 | 0.425 | 1.72E-05 |
|  |  |  |  |  | Superior Frontal | 0.359 | 0.274 | 0.444 | 8.91E-17 | 0.218 | -0.175 | 0.611 | 3.27E-01 | 0.364 | 0.204 | 0.523 | 7.81E-06 | 0.446 | 0.215 | 0.677 | 9.19E-03 | 0.364 | 0.231 | 0.497 | 8.39E-08 |
|  |  |  |  |  | Pars Orbitalis | 0.342 | 0.193 | 0.490 | 6.60E-06 | 0.071 | -0.363 | 0.504 | 7.65E-01 | 0.325 | 0.129 | 0.521 | 1.12E-03 | 0.377 | 0.055 | 0.699 | 7.01E-02 | 0.353 | 0.204 | 0.502 | 3.39E-06 |
|  |  |  | Temporal | surface area | Superior Temporal | 0.299 | 0.189 | 0.410 | 1.05E-07 | 0.085 | -0.325 | 0.494 | 7.00E-01 | 0.283 | 0.116 | 0.451 | 9.23E-04 | 0.144 | -0.137 | 0.425 | 3.49E-01 | 0.307 | 0.178 | 0.436 | 3.12E-06 |
|  |  |  |  |  | Banks of the Superior Temporal Sulcus | 0.325 | 0.190 | 0.460 | 2.23E-06 | 0.207 | -0.292 | 0.706 | 4.47E-01 | 0.328 | 0.153 | 0.502 | 2.42E-04 | 0.283 | -0.006 | 0.572 | 9.62E-02 | 0.337 | 0.203 | 0.471 | 8.68E-07 |
| Total brain | surface area |  | infant HC | | | 0.340 | 0.196 | 0.485 | 3.81E-06 | 0.361 | -0.231 | 0.953 | 2.63E-01 | 0.228 | 0.023 | 0.433 | 2.96E-02 | 0.129 | -0.249 | 0.508 | 5.18E-01 | 0.351 | 0.200 | 0.502 | 5.12E-06 |
| Frontal | surface area | Rostral Anterior Cingulate |  |  |  | 0.195 | 0.076 | 0.314 | 1.27E-03 | 0.181 | -0.263 | 0.624 | 4.60E-01 | 0.127 | -0.076 | 0.330 | 2.19E-01 | 0.107 | -0.190 | 0.404 | 5.08E-01 | 0.199 | 0.035 | 0.362 | 1.71E-02 |
|  |  | Caudal Anterior Cingulate |  |  |  | 0.231 | -0.008 | 0.471 | 5.86E-02 | -0.452 | -1.266 | 0.362 | 3.56E-01 | 0.096 | -0.172 | 0.364 | 4.84E-01 | 0.060 | -0.304 | 0.424 | 7.61E-01 | 0.242 | 0.049 | 0.436 | 1.42E-02 |
|  |  | Superior Frontal |  |  |  | 0.171 | 0.032 | 0.309 | 1.56E-02 | -0.018 | -0.480 | 0.445 | 9.46E-01 | 0.137 | -0.099 | 0.374 | 2.55E-01 | 0.110 | -0.189 | 0.409 | 5.12E-01 | 0.173 | -0.035 | 0.381 | 1.03E-01 |
|  |  | Pars Orbitalis |  |  |  | 0.110 | -0.023 | 0.243 | 1.05E-01 | 0.026 | -0.434 | 0.486 | 9.15E-01 | 0.050 | -0.145 | 0.245 | 6.16E-01 | -0.018 | -0.321 | 0.285 | 9.10E-01 | 0.113 | -0.044 | 0.269 | 1.58E-01 |
| Temporal | surface area | Superior Temporal |  |  |  | 0.438 | 0.304 | 0.572 | 1.50E-10 | 1.242 | 0.532 | 1.953 | 9.01E-03 | 0.479 | 0.297 | 0.661 | 2.42E-07 | 0.570 | 0.233 | 0.908 | 9.07E-03 | 0.451 | 0.311 | 0.591 | 2.99E-10 |
|  |  | Banks of the Superior Temporal Sulcus |  |  |  | 0.319 | 0.008 | 0.629 | 4.44E-02 | -0.388 | -1.312 | 0.535 | 4.96E-01 | 0.335 | 0.049 | 0.622 | 2.17E-02 | 0.456 | -0.058 | 0.970 | 1.80E-01 | 0.334 | 0.096 | 0.573 | 6.08E-03 |

| **Table S15. IVs for sensitivity analyses using a more stringent instrument selection threshold (*P*<1e-6)** | | | | | | | |
| --- | --- | --- | --- | --- | --- | --- | --- |
| **Exposure** | | | **Outcome** | | | **No. of instrumental variables** | **Instrumental variables** |
| infant HC | | | cEF | | | 9 | rs10846487,rs143094271,rs4674101,rs6016511,rs62580922,rs7658192,rs7792211,rs8756,rs9853018 |
| Total brain | surface area |  | cEF | | | 10 | rs10747968,rs10878349,rs12452834,rs12630663,rs2195243,rs284526,rs325700,rs41277471,rs72959469,rs7975351 |
| Frontal | surface area | Medial Orbitofrontal |  |  |  | 4 | rs12624716,rs41277471,rs7312464,rs7778997 |
|  |  | Rostral Anterior Cingulate |  |  |  | 7 | rs2971655,rs41277471,rs7195695,rs7531629,rs76501145,rs7778997,rs9427061 |
|  |  | Caudal Anterior Cingulate |  |  |  | 5 | rs12452834,rs10878349,rs2271386,rs2509765,rs12096763 |
|  |  | Superior Frontal |  |  |  | 4 | rs10788183,rs10878349,rs2195243,rs77037886 |
|  |  | Pars Orbitalis |  |  |  | 7 | rs10446454,rs10802064,rs10932876,rs11114027,rs2271386,rs7312464,rs74700822 |
| Parietal | surface area | Precuneus |  |  |  | 10 | rs10188396,rs10792025,rs11111293,rs1325995,rs61996723,rs660633,rs7209131,rs7601767,rs78419480,rs858993 |
|  |  | Superior Parietal |  |  |  | 14 | rs10032097,rs10109434,rs112193068,rs1163075,rs1512818,rs1978302,rs41402,rs4455881,rs4838960,rs4924345,rs6720027,rs67328311,rs7635829,rs999724 |
|  |  | Posterior Cingulate |  |  |  | 5 | rs58691096,rs6952255,rs73176834,rs7601767,rs76501145 |
| Temporal | surface area | Entorhinal |  |  |  | 5 | rs1807198,rs247786,rs62173537,rs6791514,rs746729 |
|  |  | Superior Temporal |  |  |  | 8 | rs10878349,rs113542967,rs12694390,rs1561369,rs34464850,rs6555938,rs76650567,rs8181632 |
|  |  | Banks of the Superior Temporal Sulcus |  |  |  | 5 | rs9669278,rs35597264,rs2237430,rs11710049,rs682780 |
| infant HC | | | Total brain | surface area |  | 8 | rs10846487,rs143094271,rs4674101,rs6016511,rs62580922,rs7658192,rs7792211,rs9853018 |
|  |  |  | Frontal | surface area | Rostral Anterior Cingulate | 8 | rs10846487,rs143094271,rs4674101,rs6016511,rs62580922,rs7658192,rs7792211,rs9853018 |
|  |  |  |  |  | Caudal Anterior Cingulate | 8 | rs10846487,rs143094271,rs4674101,rs6016511,rs62580922,rs7658192,rs7792211,rs9853018 |
|  |  |  |  |  | Superior Frontal | 7 | rs10846487,rs143094271,rs6016511,rs62580922,rs7658192,rs7792211,rs9853018 |
|  |  |  |  |  | Pars Orbitalis | 6 | rs143094271,rs6016511,rs62580922,rs7658192,rs7792211,rs9853018 |
|  |  |  | Temporal | surface area | Superior Temporal | 8 | rs10846487,rs143094271,rs4674101,rs6016511,rs62580922,rs7658192,rs7792211,rs9853018 |
|  |  |  |  |  | Banks of the Superior Temporal Sulcus | 8 | rs10846487,rs143094271,rs4674101,rs6016511,rs62580922,rs7658192,rs7792211,rs9853018 |
| Total brain | surface area |  | infant HC | | | 11 | rs10747968,rs12452834,rs12630663,rs2195243,rs284526,rs284849,rs325700,rs41277471,rs72959469,rs7477335,rs7975351 |
| Frontal | surface area | Rostral Anterior Cingulate |  |  |  | 7 | rs1921610,rs2971655,rs41277471,rs7195695,rs76501145,rs7778997,rs9427061 |
|  |  | Caudal Anterior Cingulate |  |  |  | 5 | rs12096763,rs12452834,rs2509765,rs6675870,rs7601767 |
|  |  | Superior Frontal |  |  |  | 5 | rs116623451,rs144513199,rs2195243,rs77037886,rs9909861 |
|  |  | Pars Orbitalis |  |  |  | 8 | rs10446454,rs10802064,rs10932876,rs11114027,rs113931321,rs1243189,rs6865689,rs74700822 |
| Temporal | surface area | Superior Temporal |  |  |  | 10 | rs113542967,rs1163249,rs12694390,rs1561369,rs2969,rs34059176,rs34464850,rs6555938,rs76650567,rs8181632 |
|  |  | Banks of the Superior Temporal Sulcus |  |  |  | 4 | rs12101308,rs2237430,rs3935357,rs682780 |

| **Table S16. Sensitivity analysis results (heterogeneity and pleiotropy tests) using a more stringent instrument selection threshold (*P*<1e-6)** | | | | | | | | |
| --- | --- | --- | --- | --- | --- | --- | --- | --- |
| **Exposure** | | | **Outcome** | | | **MR-PRESSO global test (*P-*value)** | **Cochran’s Q (*P-*value)** | **MR-Egger intercept (*P-*value)** |
| infant HC | | | cEF | | | 0.697 | 0.625 | 0.655 |
| Total brain | surface area |  | cEF | | | 0.291 | 0.243 | 0.806 |
| Frontal | surface area | Medial Orbitofrontal |  |  |  | 0.234 | 0.162 | 0.159 |
|  |  | Rostral Anterior Cingulate |  |  |  | 0.464 | 0.410 | 0.931 |
|  |  | Caudal Anterior Cingulate |  |  |  | 0.286 | 0.189 | 0.120 |
|  |  | Superior Frontal |  |  |  | 0.276 | 0.217 | 0.192 |
|  |  | Pars Orbitalis |  |  |  | 0.280 | 0.205 | 0.674 |
| Parietal | surface area | Precuneus |  |  |  | 0.278 | 0.252 | 0.901 |
|  |  | Superior Parietal |  |  |  | 0.548 | 0.508 | 0.610 |
|  |  | Posterior Cingulate |  |  |  | 0.191 | 0.157 | 0.133 |
| Temporal | surface area | Entorhinal |  |  |  | 0.339 | 0.258 | 0.309 |
|  |  | Superior Temporal |  |  |  | 0.230 | 0.156 | 0.608 |
|  |  | Banks of the Superior Temporal Sulcus |  |  |  | 0.801 | 0.698 | 0.283 |
| infant HC | | | Total brain | surface area |  | 0.185 | 0.085 | 0.537 |
|  |  |  | Frontal | surface area | Rostral Anterior Cingulate | 0.478 | 0.437 | 0.548 |
|  |  |  |  |  | Caudal Anterior Cingulate | 0.359 | 0.308 | 0.791 |
|  |  |  |  |  | Superior Frontal | 0.859 | 0.790 | 0.494 |
|  |  |  |  |  | Pars Orbitalis | 0.387 | 0.281 | 0.266 |
|  |  |  | Temporal | surface area | Superior Temporal | 0.578 | 0.516 | 0.324 |
|  |  |  |  |  | Banks of the Superior Temporal Sulcus | 0.372 | 0.283 | 0.646 |
| Total brain | surface area |  | infant HC | | | 0.446 | 0.391 | 0.945 |
| Frontal | surface area | Rostral Anterior Cingulate |  |  |  | 0.732 | 0.733 | 0.949 |
|  |  | Caudal Anterior Cingulate |  |  |  | 0.216 | 0.146 | 0.188 |
|  |  | Superior Frontal |  |  |  | 0.786 | 0.738 | 0.443 |
|  |  | Pars Orbitalis |  |  |  | 0.594 | 0.596 | 0.719 |
| Temporal | surface area | Superior Temporal |  |  |  | 0.428 | 0.337 | 0.054 |
|  |  | Banks of the Superior Temporal Sulcus |  |  |  | 0.198 | 0.120 | 0.259 |
