# Supplementary material for: Early‑life head circumference is causally associated with adult executive function through cortical surface area expansion
Source: Dev Cogn Neurosci. 2026 Aug 29;81:101805. doi: 10.1016/j.dcn.2026.101805 (PMC13571153; doi:10.1016/j.dcn.2026.101805)
Supplement: Supplementary file 2 [file mmc2.docx]

| **Supplementary Figures** | |
| --- | --- |
| Figure S1 | MR plots for the genetically predicted effect of infant HC on cEF |
| Figure S2 | MR plots for the genetically predicted effect of cEF on infant HC |
| Figure S3 | MR plots for the genetically predicted effect of total brain SA on cEF. |
| Figure S4 | MR plots for the genetically predicted effect of Frontal SA on cEF |
| Figure S5 | MR plots for the genetically predicted effect of Pareital SA on cEF |
| Figure S6 | MR plots for the genetically predicted effect of Temporal SA on cEF |
| Figure S7 | MR plots for the genetically predicted effect of cEF on total brain SA |
| Figure S8 | MR plots for the genetically predicted effect of cEF on Frontal SA. |
| Figure S9 | MR plots for the genetically predicted effect of cEF on Pareital SA. |
| Figure S10 | MR plots for the genetically predicted effect of cEF on Temporal SA |
| Figure S11 | MR plots for the genetically predicted effect of infant HC on total brain SA |
| Figure S12 | MR plots for the genetically predicted effect of infant HC on Frontal SA. |
| Figure S13 | MR plots for the genetically predicted effect of infant HC on Temporal SA |
| Figure S14 | MR plots for the genetically predicted effect of total brain SA on infant HC |
| Figure S15 | MR plots for the genetically predicted effect of Frontal SA on infant HC |
| Figure S16 | MR plots for the genetically predicted effect of Temporal SA on infant HC |
| Figure S17 | MR plots for sensitivity analyses using a more stringent instrument selection threshold (*P* < 1e-6). |

Figure S1. MR plots for the genetically predicted effect of infant HC on cEF. (a) Leave-one-out plot. (b) Forest plot. (c) Scatter plot. (d) Funnel plot.

Figure S2. MR plots for the genetically predicted effect of cEF on infant HC. (a) Leave-one-out plot. (b) Forest plot. (c) Scatter plot. (d) Funnel plot.

Figure S3. MR plots for the genetically predicted effect of total brain SA on cEF. (a) Leave-one-out plot. (b) Forest plot. (c) Scatter plot. (d) Funnel plot.

Figure S4. MR plots for the genetically predicted effect of Frontal SA on cEF. (a) Leave-one-out plot. (b) Forest plot. (c) Scatter plot. (d) Funnel plot.

Figure S5. MR plots for the genetically predicted effect of Pareital SA on cEF. (a) Leave-one-out plot. (b) Forest plot. (c) Scatter plot. (d) Funnel plot.

Figure S6. MR plots for the genetically predicted effect of Temporal SA on cEF. (a) Leave-one-out plot. (b) Forest plot. (c) Scatter plot. (d) Funnel plot.

Figure S7. MR plots for the genetically predicted effect of cEF on total brain SA. (a) Leave-one-out plot. (b) Forest plot. (c) Scatter plot. (d) Funnel plot.

Figure S8. MR plots for the genetically predicted effect of cEF on Frontal SA. (a) Leave-one-out plot. (b) Forest plot. (c) Scatter plot. (d) Funnel plot.

Figure S9. MR plots for the genetically predicted effect of cEF on Pareital SA. (a) Leave-one-out plot. (b) Forest plot. (c) Scatter plot. (d) Funnel plot.

Figure S10. MR plots for the genetically predicted effect of cEF on Temporal SA. (a) Leave-one-out plot. (b) Forest plot. (c) Scatter plot. (d) Funnel plot.

Figure S11. MR plots for the genetically predicted effect of infant HC on total brain SA. (a) Leave-one-out plot. (b) Forest plot. (c) Scatter plot. (d) Funnel plot.

Figure S12. MR plots for the genetically predicted effect of infant HC on Frontal SA. (a) Leave-one-out plot. (b) Forest plot. (c) Scatter plot. (d) Funnel plot.

Figure S13. MR plots for the genetically predicted effect of infant HC on Temporal SA. (a) Leave-one-out plot. (b) Forest plot. (c) Scatter plot. (d) Funnel plot.

Figure S14. MR plots for the genetically predicted effect of total brain SA on infant HC. (a) Leave-one-out plot. (b) Forest plot. (c) Scatter plot. (d) Funnel plot.

Figure S15. MR plots for the genetically predicted effect of Frontal SA on infant HC. (a) Leave-one-out plot. (b) Forest plot. (c) Scatter plot. (d) Funnel plot.

Figure S16. MR plots for the genetically predicted effect of Temporal SA on infant HC. (a) Leave-one-out plot. (b) Forest plot. (c) Scatter plot. (d) Funnel plot.

Figure S17. MR plots for sensitivity analyses using a more stringent instrument

selection threshold (*P* < 1e-6).(a) Leave-one-out plot. (b) Forest plot. (c) Scatter plot. (d) Funnel plot.
